# Synthesis of physically crosslinked PAM/CNT flakes nanocomposite hydrogel films *via* a destructive approach†

**DOI:** 10.1039/d1ra07825a

**Published:** 2021-12-07

**Authors:** Alireza Yaghoubi, Ali Ramazani, Hossein Ghasemzadeh

**Affiliations:** Department of Chemistry, Faculty of Science, University of Zanjan 45371-38791 Zanjan Iran aliramazani@gmail.com aliramazani@znu.ac.ir; Department of Biotechnology, Research Institute of Modern Biological Techniques (RIMBT), University of Zanjan 45371-38791 Zanjan Iran; Department of Chemistry, Faculty of Science, Imam Khomeini International University Qazvin 34148-96818 Iran

## Abstract

Carbon nanotube (CNT)-based hydrogels have recently found a wide variety of applications due to the unique physical and chemical properties of CNTs. CNTs can be used as a nanofiller and/or crosslinker to produce nanocomposite hydrogels with good mechanical and structural properties. In this research, a novel method was reported for producing polyacrylamide (PAM)/oxidized-multiwalled carbon nanotube (O-MWCNT) flakes nanocomposite hydrogel films without using any organic cross-linker or surfactant. Through a mechanism dependent on the reactive oxygen species (ROS), some O-MWCNTs were broken down *in situ* into small flakes in the aqueous solutions containing acrylamide (AM) and sodium persulfate (NaPS) at the temperature range of 85–90 °C. Simultaneously, *in situ* polymerization of the AM monomers occurred using free radicals, which resulted in the formation of PAM chains. The flakes acted as crosslinkers by forming hydrogen bonds with PAM chains and formed a hydrogel network after 48 h at room temperature. The hydrogels were characterized by different techniques (FT-IR, Raman, FE-SEM, TEM, TGA, tensile test). The porous structure of the hydrogel films as well as micro-network structures with unique morphologies were observed by SEM. The O-MWCNT flakes and some undegraded O-MWCNTs in the hydrogel network were also observed by TEM. The results showed that PC_2_I_2_H hydrogel film, as an evolved hydrogel, has excellent swelling performance in aqueous solutions at different pH and temperatures. In addition, this hydrogel showed a tensile strength of 103 MPa in the dry state and an elongation of 703% in the swollen state.

## Abstract 2

Novel PAM/CNT flakes nanocomposite hydrogel films were synthesized by *in situ* degradation of the oxidized-MWCNTs into flakes using persulfate activation. The flakes crosslinked the PAM chains *via* hydrogen bonding to form a hydrogel network.

## Graphical abstract

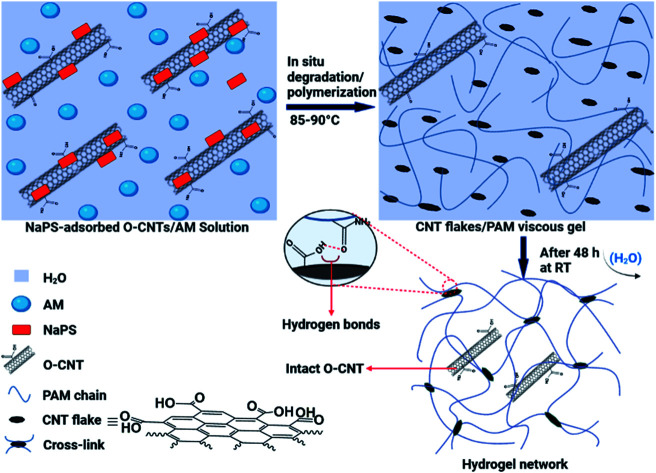

## 1 Introduction

Hydrogels are hydrophilic polymeric networks with the ability to absorb and retain large amounts of water. These soft materials with high biocompatibility have been applied for various purposes, especially in biological field.^1–4^ Recently, hydrogels have found wide applications in many fields, such as smart control equipment,^5^ body motion sensors,^6^ catalyst,^7^ water/oil separation filter,^8^ flexible electronics,^9^ and controlling contaminants in wastewater.^10^ Among them, CNT-based nanocomposite hydrogel films, as a new class of hydrogels, have attracted much interest^11,12^ due to the excellent properties of CNTs arising from their unique π-conjugated structure. CNTs have high electrical and thermal conductivity, high area surface, as well as extraordinary optical, chemical, mechanical properties. CNT-based hydrogel films have emerged for a variety of applications such as cardiac tissue engineering,^13^ electronic devices,^12^ tissue engineering,^14^ sensors^15^ and the energy storage devices.^11^ Various methods have been reported for the synthesis of CNT-based hydrogel films. Kovtyukhova *et al.* prepared individual oxidized tubes by oxidative exfoliation of SWCNT ropes. The oxidized tubes could form viscous hydrogels at low concentration due to the formation of a hydrogen-bonded nanotube network. The oxidized SWCNTs easily bonded to amine-coated surfaces to form monolayer films.^16^ Chen *et al.* prepared SWCNT hydrogels by adding CaCl_2_ to stable SWCNT dispersions. Then, through a phase change method, eicosane was infiltrated into SWCNT scaffolds and flexible films were produced for thermal energy storage.^17^ Using a simple process of vacuum-filtration, Chen *et al.* produced a highly conductive hydrogel film composed of chitin nanofibers and MWCNTs. This hydrogel film showed potential application in foldable electronic devices.^18^ In another approach, CNT films synthesized by chemical vapor deposition (CVD) method were first activated electrochemically. Then, polyaniline was decorated around CNTs to fabricate a hydrogel film. This film, as a supercapacitor, exhibited great potential in the field of energy storage devices.^11^

The long-term toxicity of CNTs, which is mostly due to their length,^19^ has led to a lack of development of the CNT-based hydrogels and their limitation for use in living systems. Degradation of CNTs is an effective way to reduce the toxicity of CNTs. Various studies have shown that CNTs can be degraded by various biological agents such as macrophages,^20,21^ enzymes^22,23^ and bacteria.^24^ Oxidation processes in bio-degraders produce reactive oxygen species (ROS) such as hydrogen peroxide (H_2_O_2_) and superoxide anion (O_2_˙^−^). ROS play a vital role in biodegradation of CNTs *via* a process called oxidative burst (the rapid release of ROS from different types of cells).^25^ For example, the interaction of H_2_O_2_, as a strong oxidant, with the active center of horseradish peroxidase, can produce extremely strong oxidizing species, resulting in a reduction in CNTs length.^26^ In the other hand, chemical degradation is mainly based on the use of some strong oxidizing agents, which can change the physicochemical properties of CNTs due to the occurrence of oxidation reactions.^23,27,28^ For example, during chemical degradation by O_3_ treatment, the π-conjugated structure of SWCNTs is removed and a large number of oxygen-containing groups are formed on the SWCNT surfaces.^28^

The use of degraded CNTs (flakes) for the synthesis of hydrogels can have several advantages as follows: by controlling the degradation of CNTs, the CNTs-induced cytotoxicity for biological applications can be reduced;^19^ CNT flakes can act as physical crosslinkers through hydrogen bonding interactions due to their high oxygen-containing groups, in which case toxic organic crosslinkers are not used; by controlling the different sizes of CNT flakes (as crosslinkers), hydrogels with various properties can be produced; and it is a novel method of degradation and/or synthesis with a valuable product.

In this study, we report for the first time a facile and novel technique for synthesis of the PAM/O-MWCNT flakes nanocomposite hydrogel films using O-MWCNTs and NaPS *via* a destructive approach without using any cross-linkers or surfactants. In this way, we developed a novel method for the synthesis of CNT-based hydrogels. In this method, the flakes produced from degradation of O-MWCNTs could act as crosslinkers for hydrogel film formation by forming hydrogen bonds with the PAM chains. The hydrogel films were characterized by different techniques and their morphology was thoroughly examined. Swelling behaviour was systematically investigated at different pH and temperatures. In addition, their mechanical strength was also evaluated. The evolved PC_2_I_2_H hydrogel film showed the highest thermal stability and mechanical strength compared with other hydrogels.

## 2 Experimental

### 2.1 Materials

Acrylamide (AM, 99%), sodium persulfate (NaPS, 98%), nitric acid (HNO_3_,70%) and sulfuric acid (H_2_SO_4_, 98%) were purchased from Sigma-Aldrich and used as received. Multi-walled carbon nanotube (MWCNT) was purchased from EXIR Co. (Wein, Austria). The MWCNTs had an outer diameter of 5–15 nm, lengths in the range of 10–30 μm and purity >95%.

### 2.2 Oxidation of pristine MWCNTs

Pristine MWCNTs were oxidized according to a reported method^29^ with a slight change in the method. In a typical procedure, 0.5 g of pristine MWCNT was first treated by 200 mL of the mixed acid (H_2_SO_4_/HNO_3_) (3 : 1, v/v) under sonication in an ultrasonic bath for 4 h at 40 °C and subsequent sonication with a probe for another 1 h. Then, the resultant suspension was diluted with 1000 mL of distilled water and followed by centrifugation. Finally, the product was filtered and washed repeatedly with distilled water until neutral pH was reached and then dried in a vacuum oven at 70 °C for 24 h to obtain O-MWCNTs. The oxidation of the MWCNTs was confirmed by FTIR, TGA and Raman (Fig. S1, S2† and 3a, respectively). In addition, the O-MWCNTs dispersed into distilled water were able to form highly stable suspensions under ambient conditions (Fig. S3†).

### 2.3 Synthesis of the PAM/CNT flakes nanocomposite hydrogel films

First, a certain amount of O-MWCNTs (0.0005,0.001 and 0.002 g) was poured into distilled water (7 mL) and subjected to ultrasonic waves by using an ultrasonic probe for 45 min to obtain a homogeneous suspension of the O-MWCNTs. Then, the aqueous suspension was deoxygenated by bubbling with nitrogen gas for 1 h and transferred to a 50 mL round bottom flask equipped with a magnetic stirrer and a nitrogen gas inlet tube at room temperature. After about 5 min of vigorous stirring, a designated amount of NaPS (0.001 and 0.002 g, dissolved in 1 mL of distilled water) was added to the prepared suspension and stirred at 500 rpm for 10 min to adsorb the NaPS on the walls of the nanotubes. After that, a constant amount of AM monomers (1.2 g, dissolved in 2 mL of distilled water) was added to the previous suspension and stirring at 375 rpm was continued for another 20 min to achieve better dispersion of the components. Finally, by increasing the temperature of the reaction mixture to an average value of about 87.5 °C, *in situ* degradation/polymerization was initiated and after about 40 min of stirring a viscous black mixture was obtained. After cooling to room temperature, the product was poured into a Petri dish and allowed to dry at room temperature for 48 h to obtain a thin hydrogel film. Parts of the preparation process of the hydrogel films are shown in Fig. S6 and Video S1.†

Table 1 shows the required raw materials and conditions for the formation of the hydrogel films, in which the samples are expressed as P_*x*_C_*m*_I_*n*_H or P_*x*_C_*m*_I_*n*_ codes, where P, C, I and H stand for PAM, O-MWCNTs, NaPS, and hydrogel, respectively, as well as *x*, *m* and *n* represent the feed amounts of AM monomer, O-MWCNT and NaPS, respectively. For example, the PC_0.5_I_1_H implies that 0.5 × 10^−3^ g O-MWCNT, 1 × 10^−3^ g NaPS and 1.2 g AM (a constant amount for all used codes) are the feed amounts in the synthesis of the hydrogel films. In Table 1, based on the results of the swelling studies (Fig. S4, S5 and Table S1†), the optimal mass ratio of NaPS to O-MWCNT was obtained for the formation of optimal hydrogel films with good swelling properties (Table S2†).

**Table 1:** The composition of the feed material for the synthesis of PAM/O-MWCNT flakes nanocomposite hydrogel films^a^

Sample	AM (g)	NaPS (g)	O-MWCNT (g)	H_2_O (mL)	Results
PC_0.5_I_0.5_	1.2	0.0005	0.0005	10	NR.
PC_0.5_I_1_H^b^	1.2	0.001	0.0005	10	**H. film**
PC_1_I_1_H	1.2	0.001	0.001	10	**H. film**
PC_2_I_2_H	1.2	0.002	0.002	10	**H. film**
PC_3_I_3_	1.2	0.003	0.003	10	Film
PC_4_I_4_	1.2	0.004	0.004	10	Film

a NR., no reaction; H. film, hydrogel film.

b Optimal hydrogel was formed only at a weight ratio of 2 to 1 (NaPS to O-MWCNT).

### 2.4 Swelling studies

Swelling experiments were performed by immersing a certain amount of as-synthesized hydrogel films into distilled water in different conditions with the following purposes: (1) Optimizing the feed amount of NaPS for synthesis of the hydrogel films (Fig. S4, S5 and Table S1†); (2) Investigating the swelling performance of the hydrogel films at different pH and temperatures. At different intervals, the swollen hydrogels were taken from the water and weighed after removing excess water from their surface. The swelling ratio of different hydrogels was evaluated using the (W_s_ − *W*_d_)/*W*_d_ equation, where *W*_s_ and *W*_d_ are the weight of the swollen and dry hydrogels, respectively.

### 2.5 Characterization

Fourier transform infrared (FTIR) spectra of the hydrogel films were recorded on a Thermo Nicolet Avatar 380 FTIR (USA) using a smart orbit diamond ATR (the attenuated total reflectance) in the wavenumber range of 4000–600 cm^−1^. In addition, FTIR spectra of the MWCNTs and O-MWCNTs were obtained in the range of 4000–400 cm^−1^. The Raman spectra were collected using a TakRam N1-541 spectrometer (Iran) equipped with a Hamamatsu detector. The 532 nm laser was used to excite the samples. The baseline correction was performed for all spectra and fitted by origin software in the range from 4000 to 400 cm^−1^. The morphology of the hydrogel films was characterized using the field emission scanning electron microscope (FE-SEM) (TESCAN MIRA 3, Czech Republic) with an accelerating voltage of 10 kV. The fracture surfaces were coated with a thin layer of gold by sputter coating method. The morphology of the PC_2_I_2_H hydrogel film and O-MWCNTs were characterized by the transmission electron microscopy (TEM, Philips EM208S, The Netherlands) at an accelerating voltage of 100 kV. The film specimen was first embedded in epoxy resin to produce resin blocks. The specimen block was then trimmed and obtained the sections with approximately dimensions of 1.2 mm by 1.2 mm. Subsequently, the trimmed specimen was placed into the ultra-microtome and cut with a diamond knife to obtain ultrathin sections about 70 nm and finally collected on a copper mesh TEM grid. For O-MWCNTs, the suspension of O-MWCNTs was dropped on a copper mesh grid and allowed to dry before TEM analysis. Thermal stability of the samples was measured by thermogravimetric analysis (TGA, SDT Q600, USA) from room temperature to 750 °C at a heating rate of 10 °C min^−1^ under N_2_ atmosphere. The weight of the samples was in the range of 3–5 mg. The tensile tests of the hydrogel films were measured using a Universal Material Testing Machine (Hounsfield-H10Ks, USA) equipped with a 500 N load cell at room temperature. As-synthesized hydrogel films were cut in dumbbell shaped samples, with a gauge length of 15 mm, a width of 10 mm, and a thickness of 0.32 mm. The tensile tests of these hydrogels were conducted at a crosshead rate of 5 mm min^−1^. On the other hand, the measurements of the hydrogel samples in the swollen state were done as size of ∼15 mm in gauge length, ∼32 mm in width, and a ∼1.12 mm in thickness. In addition, the crosshead rate was set as 5 mm min^−1^. The tensile tests were performed in triplicate for all hydrogel film samples. According to the stress–strain curves, the Young's modulus was calculated from the slope of the linear region (in the strain range of the 2–6% and 0–200% for as-synthesized and swollen hydrogel films, respectively). The tensile strength was calculated as the maximum load divided by the original cross-sectional area of the sample. In addition, the elongation at break was defined as ratio between increased sample length at breaking point and its original length.

## 3 Results and discussion

### 3.1 Synthesis and formation mechanism of the nanocomposite hydrogel films

**Fig. 1:**
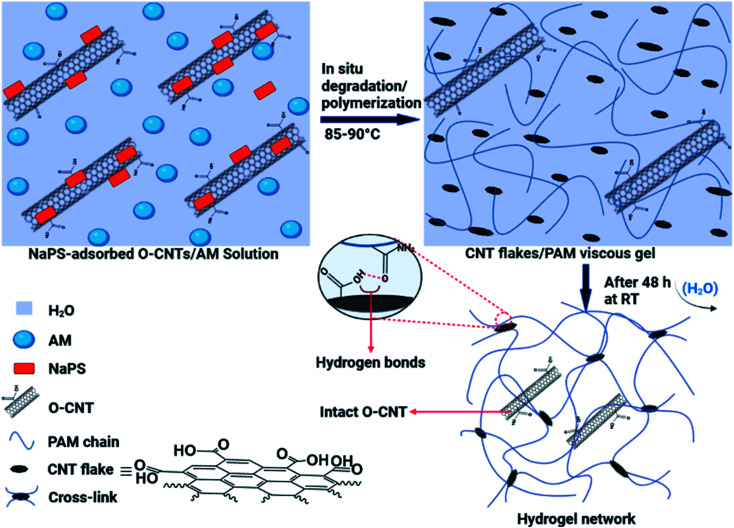
Schematic illustration of the synthetic process of the hydrogel films and interactions involved in the network of the hydrogel.

The *in situ* degradation of some O-MWCNTs in the aqueous solution of AM and simultaneously, free radical polymerization of AM monomers produced a viscous suspension, including the flakes with different sizes and the PAM chains (Fig. 1). Next, a network of the hydrogel films was formed by removing water molecules from the mixture (by evaporation) and crosslinking of PAM chains through hydrogen bonding (Fig. 1). The hydrogen bonds can be formed in the hydrogel network between the oxygen-containing groups (especially COOH) on the surface of the flakes (free O-MWCNTs also contribute to hydrogen bonding) and the amide groups from PAM chains (Fig. 1). In addition to hydrogen bonds, hydrophobic interactions can also occur in the network, including interactions between the flakes and/or free O-MWCNTs and the PAM chains, as well as between PAM chains.

The proposed mechanism for the synthesis of the hydrogel films generally consists of two parts: the *in situ* degradation of some O-MWCNTs (especially nanotubes with more defects) and simultaneous *in situ* polymerization of AM monomers; and crosslinking of PAM chains through hydrogen bonding between degraded O-MWCNTs (flakes) and amide groups (from PAM) (Fig. 1). Degradation of O-MWCNTs can occur through the synergistic effect of the persulfate (NaPS) activation by the surrounding O-MWCNTs as well as heat^30^ (Fig. 1). In addition, the degradation by both radical and non-radical pathways can be done collectively with the dual role of O-MWCNTs in degradation process. In the radical pathway, the activation of the persulfate in the presence of O-MWCNTs (*via* electron transfer) as well as thermal energy, leads to the dissociation of the O–O bond in the persulfate and ultimately the production of ROS (˙OH and SO_4_˙^−^).^30–33^ The non-radical pathway is confined to the surface of the O-MWCNTs, and ROS include the surface activated complex (O-MWCNT/persulfate), surface-bound SO_4_˙^−^, and ^1^O_2_.^30^ The produced ROS in both pathways are capable of degrading various organic compounds, especially phenols.^30,31^ As shown in Fig. S1 and S2,† the O-MWCNTs contain large amounts of oxygen-containing functional groups, and the presence of conjugated cyclic structures (*e.g.*, phenol) in the O-MWCNTs is very likely. Oxidative degradation of these structures on the surfaces of O-MWCNTs can affect their electronic structure by creating new defects on the walls of O-MWCNTs, which facilitates further oxidation and/or degradation processes. In agreement with swelling experiments (Fig. S4†), the NaPS can be adsorbed on the surfaces of O-MWCNTs and trigger *in situ* degradation/polymerization.^34^ Mixing highly stable suspensions of the O-MWCNTs with NaPS under intense stirring leads to strong adsorption of the NaPS on the walls of O-MWCNTs, which provides the conditions for non-radical degradation. In this regard, defective O-MWCNTs can approach the complex of O-MWCNTs/NaPS *via* π–π interactions and/or hydrogen bonding. Then, the transfer of electrons from electron donor groups on the surfaces of O-MWCNTs (*e.g.*, the hydroxyl) to adsorbed NaPS leads to the oxidation of these groups. In addition, the generation of single oxygen (^1^O_2_) can also occur in the presence of the surface carbonyl groups as well as at O-MWCNTs defects.^32,33^ The necessity of the NaPS adsorption on the walls of O-MWCNTs for their degradation is consistent with the experimental results of the hydrogel films synthesis (Table 1). At low amounts of NaPS (0.0005 g), due to insufficient absorption of NaPS on the walls of O-MWCNTs, the degradation and subsequent hydrogel formation do not occur. In addition, at high amounts of NaPS (0.003 and 0.004 g), the lack of NaPS adsorption onto O-MWCNTs is due to the O-MWCNTs agglomeration (due to van der Waals forces), and only a polymer mixture (film) is formed by acrylamide polymerization (Table 1). In addition to the above mentioned, the formation of hydrogel with pristine MWCNTs failed and resulted in the formation of agglomerates. This is another evidence for the need to adsorb NaPS onto the O-MWCNTs for successful synthesis of the hydrogels. The higher the NaPS to O-MWCNTs ratio, the smaller the flakes due to more degradation, and the weaker the crosslinking in the network, resulting in reduced hydrogel swelling (Table S1†). This may be due to the formation of the flakes batches resulting from hydrogen bonding between very small flakes (Fig. 5f).

### 3.2 FT-IR and Raman studies

**Fig. 2:**
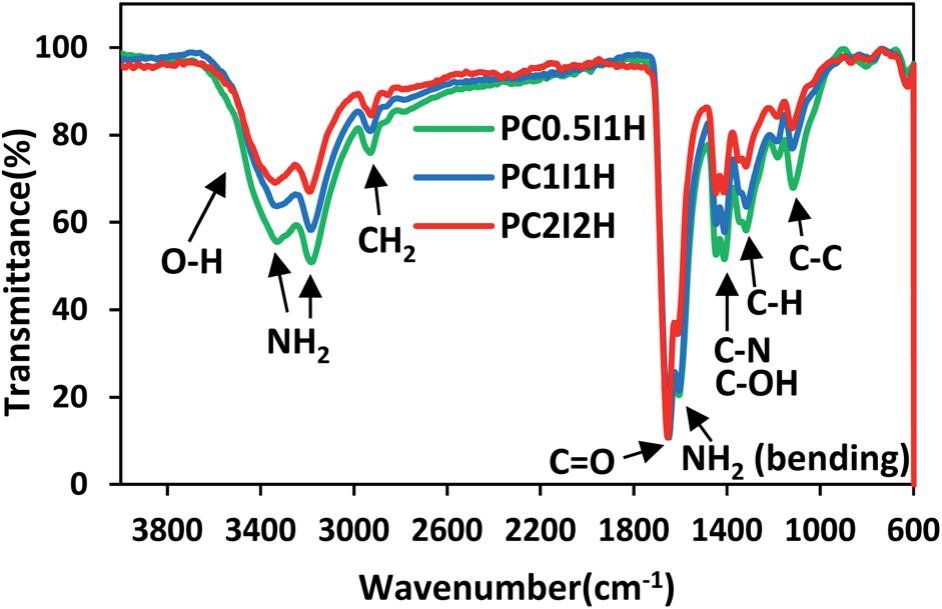
The ATR-FTIR spectra of freeze-dried hydrogel films.

The ATR-FTIR spectra of the hydrogel films in the wavenumber range of 600–4000 cm^−1^ are shown in Fig. 2. The spectral pattern of the porous hydrogel films is very similar. However, the overall intensity of the peaks in the spectra decreases with increasing the content of the flakes (crosslinkers) in the hydrogels (Fig. 2). This may be because the crosslinking density increases with increasing flakes content, which causes IR active functional groups (mainly related to the PAM chain) to be involved in crosslinking. Table S3† summarizes the types of vibrations and the position of the IR active bonds in the hydrogel films.^35,36^ The characteristic peaks of the hydrogel films in the range of 3329–3338 cm^−1^ and 3184–3190 cm^−1^ are attributed to the asymmetric and symmetric –NH_2_ stretching vibrations, respectively.^35^ The absorption peaks in the range of 2925–2930 cm^−1^ and 2853–2871 cm^−1^ can be related to the stretching vibrations of –CH_2_ and –CH groups from the PAM chains. A sharp and strong peak in the range of 1648–1656 cm^−1^ is observed, which corresponds to the C=O stretching vibration of the amide groups (from PAM) and/or carboxyl groups (from O-MWCNT flakes and free O-MWCNTs) in the hydrogel networks. In addition, the intensity of this peak in PC_2_I_2_H hydrogel is higher than other hydrogels due to the higher content of the flakes in the network. The absorption peak at 1606–1612 cm^−1^ can be attributed to the NH_2_ bending vibrations. As shown in Fig. 2, as the content of the flakes in the hydrogel films increases, a decreasing trend in the intensity of the NH_2_ bending peak in the spectra is observed. This may be because the amide groups of the hydrogel network are involved in the formation of hydrogen bonds with carboxyl groups (from O-MWCNT flakes and free O-MWCNTs). These observations can be used as evidence for the crosslinking nature of the hydrogel networks based on hydrogen bonding. In addition, the absorption peaks of the O-MWCNTs are invisible due to their overlap with the PAM chains peaks as well as the low content of free O-MWCNTs in the hydrogel networks. Other important peaks in the range of 600–1600 cm^−1^, which are often assigned to the bending vibrations,^36^ are listed in Table S3.†

**Fig. 3:**
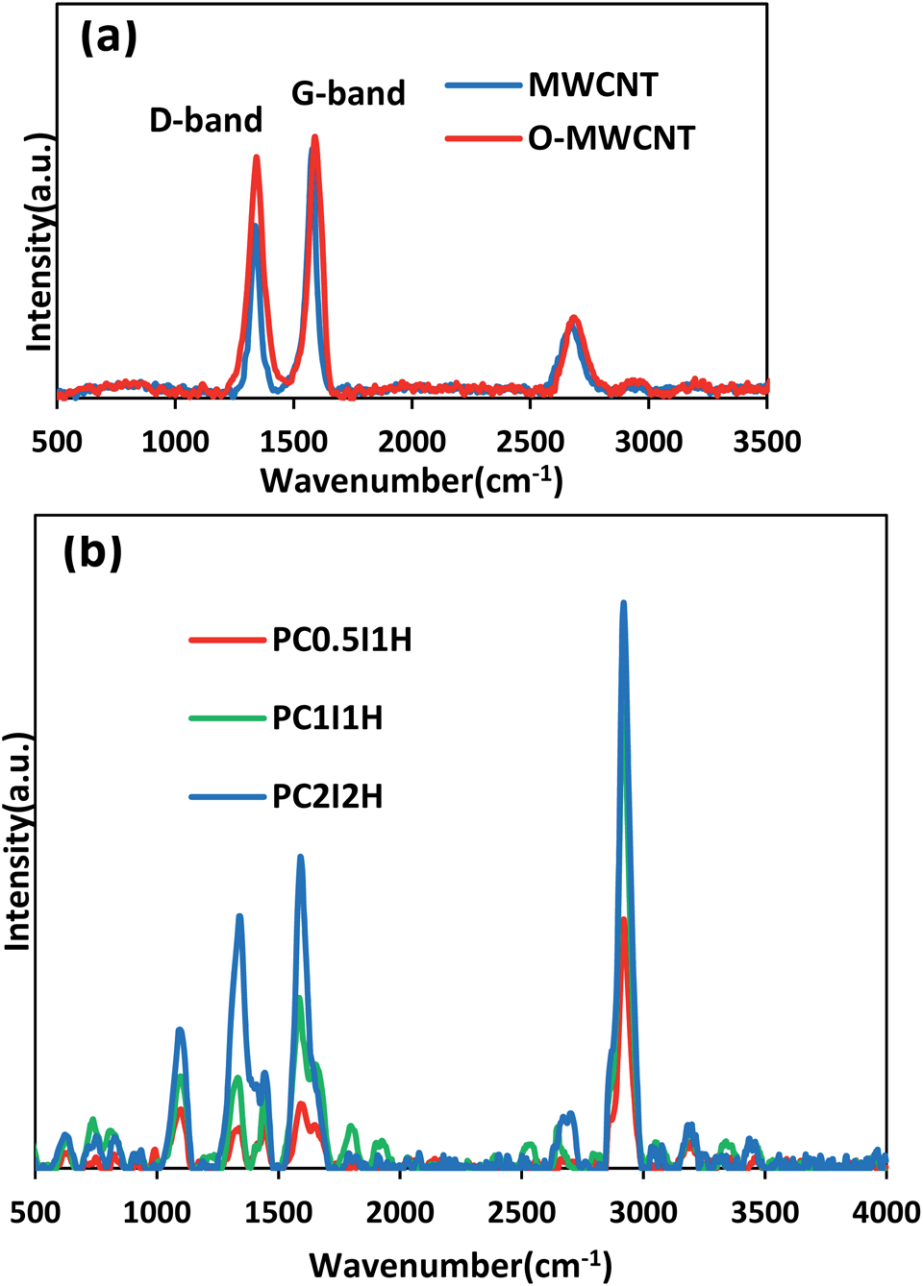
(a) Raman spectra of pristine MWCNT and O-MWCNT, (b) Main Raman spectra of freeze-dried hydrogel films.

Since Raman spectroscopy is a powerful tool for studying structural and electronic properties of CNTs.^37^ Raman analysis was applied to evaluate the structure of the MWCNTs, O-MWCNTs and the synthesized hydrogel films.

Fig. 3a shows the Raman spectra of the pristine MWCNTs and O-MWCNTs. From the Raman spectrum of pristine MWCNTs, the D-bond (the disorder in graphite structure) and the G-bond (the graphite structure) appear at 1337 and 1579 cm^−1^, respectively. After the oxidation treatment of pristine MWCNTs, the position of the peaks shifted to 1344 and 1590 cm^−1^ in the D-bond and G-bond, respectively. This may be due to less inter-tube interactions between the dispersed O-MWCNTs, leading to the shift of Raman peaks to higher wavelengths.^38^ In addition, the change in the position of the G-band in pristine MWCNTs and O-MWCNTs can be attributed to the change in the electronic structure of the nanotubes after the oxidation process.^39^ Furthermore, the intensity ratio of the D and G-bands (*I*_D_/*I*_G_) increased from 0.68 in pristine MWCNTs to 0.92 in O-MWCNTs. This confirms the introduction of defects (*i.e.*, oxygen-containing groups, especially carboxyl and hydroxyl groups) on the walls of the nanotubes after the oxidation treatment. Raman results for evaluating the oxidation of the MWCNTs are in complete agreement with the results of IR and TGA (Fig. S1 and S2,† respectively).

Fig. 3b shows the Raman spectra of the freeze-dried hydrogel films. From the Raman spectra of the hydrogel films, the bands located at 1344 and 1590 cm^−1^ can be attributed to the D-bond and G-bond (from free O-MWCNTs), respectively. In addition, the *I*_D_/*I*_G_ ratio from the Raman spectra of the hydrogel films generally shows an increasing trend with increasing the feed amount of O-MWCNT (Table 2). This may be due to the presence of coatings on the sidewalls of the nanotubes, which increases with increasing the content of degraded O-MWCNTs (flakes) in the hydrogel network.^40^ The unusual value of the *I*_D_/*I*_G_ ratio for PC_0.5_I_1_H can be attributed to residual sodium ions (coating agent) in the network. Furthermore, an increasing trend is observed in the ratio of the G-band (from the Raman spectrum of hydrogels) to the PAM characteristic peak at 2923 cm^−1^ (which is attributed to the stretching vibration of CH_2_) (Table 2). This can be attributed to the relative concentration of free O-MWCNTs in the hydrogels network. The Raman bands at 1610 cm^−1^ and 1322 cm^−1^ are related to the bending vibrations of NH_2_ and C–H, respectively (from PAM chains). These bands overlap with the D and G bands in the hydrogel films spectra. The band at 1458 cm^−1^ is assigned to the bending vibration of CH_2_ group in the PAM chain. In addition, other bands in the Raman spectra of the hydrogels at 626, 836, 1105, 3196 and 3356 cm^−1^ are related to C=O bending, C–C side-chain and C–C skeletal stretching, as well as NH_2_ symmetry and asymmetry stretching, respectively, indicating no overlap with O-MWCNT peaks (Fig. 3b). In summary, Raman studies confirmed defects created in the sidewalls of the O-MWCNTs, as well as the presence of free O-MWCNTs and O-MWCNT flakes (as coating agents) in the hydrogel network. These results are in accordance with the obtained data from FTIR analysis of the hydrogel films.

**Table 2:** Raman parameters of the hydrogel films

Samples	PC_0.5_I_1_H	PC_1_I_1_H	PC_2_I_2_H
*I*_D_/*I*_G_	0.68	0.53	0.92
*I*_G_/*I*_characteristic peak_	0.26	0.37	0.55

### 3.3 SEM analysis

**Fig. 4:**
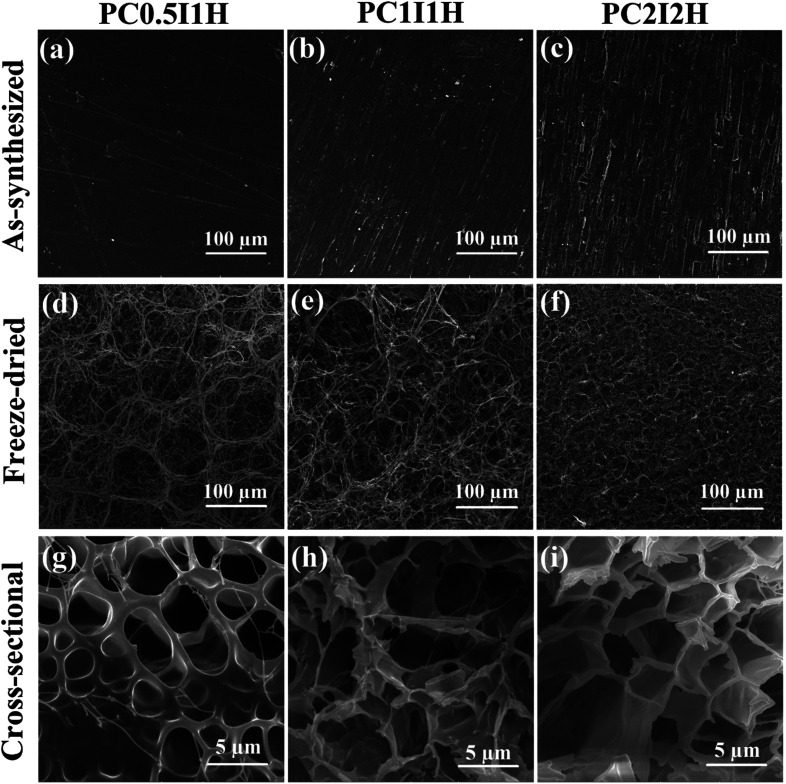
(a–f) The SEM image of the outer surface of different hydrogel films (top-view). (a–c) As-synthesized hydrogel films of PC_0.5_I_1_H, PC_1_I_1_H and PC_2_I_2_H, respectively, (d–f) freeze-dried hydrogel films of PC_0.5_I_1_H, PC_1_I_1_H, PC_2_I_2_H, respectively. (g–i) The cross-sectional SEM images of freeze-dried hydrogel films. (g) PC_0.5_I_1_H, (h) PC_1_I_1_H and (i) PC_2_I_2_H.

SEM analysis was performed to study the morphology and microstructures of different hydrogel films. Fig. 4a–f shows the SEM images of the as-synthesized and freeze-dried hydrogel films at 500× magnification. As shown in Fig. 4a–f, the hydrogel films before and after swelling in distilled water have completely different morphologies. This is mainly due to the difference in the structure of the hydrogel network in the dry (film) and wet (swollen) state. In addition, freeze drying method for the preparation of SEM hydrogel samples may also be effective in morphological differences. Fig. 4a–c shows the surface relief of as-synthesized hydrogel films in the form of parallel linear microstructures. The diameter of these microstructures increased with increasing the feed amount of O-MWCNT from 0.04 wt% in PC_0.5_I_1_H hydrogel film to 0.16 wt% in PC_2_I_2_H hydrogel film. However, these relief patterns disappeared when the feed amount of O-MWCNT was increased to 0.25 wt% in the PC_3_I_3_ film (Fig. S7†). The formation of the surface relief attests to the successful interactions between the O-MWCNT flakes and the PAM chains to form a 3D network of the hydrogel films. Whereas, the absence of the surface microstructures in the PC_3_I_3_ film (Fig. S7†) and its dissolution in distilled water without swelling, confirm the lack of crosslinking to form a hydrogel network. Fig. 4d–f shows the unique morphologies of the micro-network structures onto the outer surface of freeze-dried hydrogel films. These microstructures are observed as annular (Fig. 4d and e) and spider web-like modes (Fig. 4f). The annular patterns on the surface of the hydrogel films gradually lose their shape (as well as the thickness of branches in the micro-network structure decreases) with increasing the feed amount of O-MWCNTs (Fig. S8†). For example, the outer surface of the PC_0.5_I_1_H shows circular shapes (Fig. 4d), whereas these shapes have disappeared in the PC_2_I_2_H surface (Fig. 4f). Part of the annular and spider web-like microstructures onto the surface of freeze-dried hydrogel films are shown in Fig. S8.† The micro-network structures located on the surface of the hydrogel films are generated due to phase separation between the crosslinks and water.^41–43^ The formation of thicker micro-network structures in PC_0.5_I_1_H hydrogel compared with other hydrogels may be attributed to the high weight ratio of NaPS and/or AM to feed O-MWCNTs, which induces more phase separation (Fig. S8†). In other words, poor crosslinking in the PC_0.5_I_1_H hydrogel network may cause the annular microstructures, which are mainly made of PAM chains, to form in a separate phase. These results confirm the different intensities of the peaks in the IR spectra of different hydrogel films. The micro-network structures may increase the mechanical strength of the hydrogel films. It has previously been reported that the formation of embedded micro-network structures leads to an improvement in the mechanical properties of the hydrogels.^41–45^

**Fig. 5:**
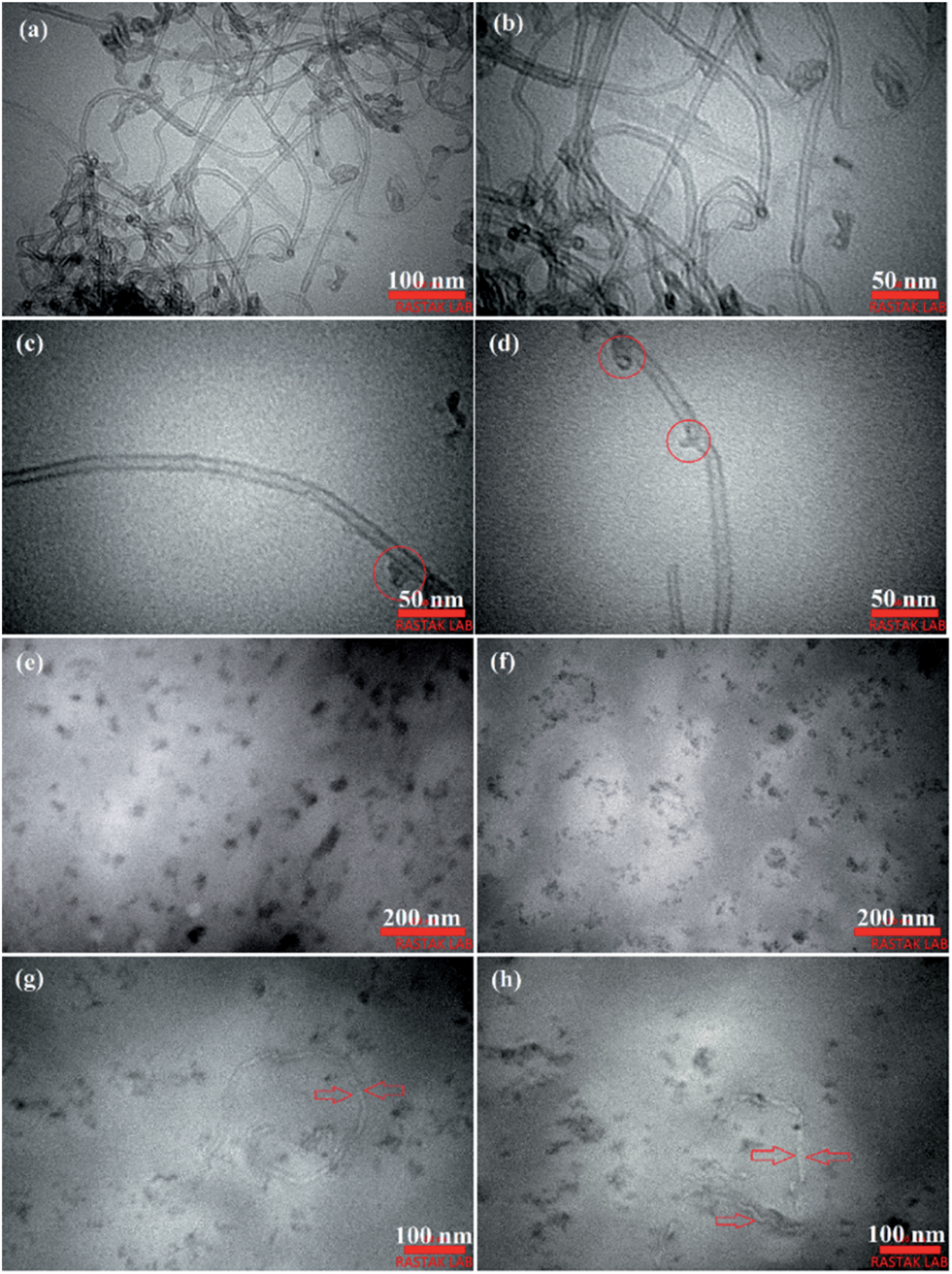
TEM images of the O-MWCNTs and as-synthesized PC_2_I_2_H film. (a) O-MWCNTs, (b) a high magnification of (a), (c and d) TEM images of a single nanotube with some marked inherent defects, (e) individual O-MWCNT flakes, (f) the flake batches, (g and h) TEM images of incompletely degraded O-MWCNTs among flakes.

As shown in Fig. S8,† the SEM micrographs of different hydrogels surfaces show the presence of a porous network under the micro-network structures at different magnifications. The cross-sectional SEM images of the porous networks in freeze-dried hydrogel films are shown in Fig. 4g–i. In contrast to the surface morphology of the hydrogel films with abundant micro-network structures, the cross-sectional morphology of the hydrogels shows only sparse micro-network structures in the PC_0.5_I_1_H sample. SEM micrographs of Fig. 4g–i show gradual changes in the structure of the hydrogel pores with increasing the content of the O-MWCNT flakes in the network. The structure of the hydrogel pores of PC_0.5_I_1_H is observed in a circular shape with a thick wall, while in PC_1_I_1_H this structure becomes irregular and finally in PC_2_I_2_H with the highest flake content, the shape of the hydrogel pores changes to a polygon with a thin wall. This may be due to the more accurate crosslinking in the PC_2_I_2_H hydrogel network than other hydrogels, which results from the appropriate content of the flakes in the hydrogel network formation. In addition, the average diameter of the pores in the hydrogels (especially PC_2_I_2_H hydrogel with a more uniform size) is about 5 μm (Fig. 4i). This indicates that the hydrogels synthesized in this work can be classified as macroporous hydrogels. Due to their interconnected structure, these types of hydrogels may find applications in drug delivery.^46^ The porous structure and microstructures of the nanocomposite hydrogel films make them very promising for use as electromagnetic composites by controlling the content of CNTs.^47–51^ Recently, carbon-based composites with porous structures have gained extensive attention as lightweight microwave absorbers.^47–50^ Therefore, it can be concluded that in the formation of the hydrogel films, an evolutionary process occurs with increasing the content of the O-MWCNT flakes (as crosslinkers). In addition, the PC_2_I_2_H hydrogel is an evolved sample of the hydrogels synthesized in this work. SEM images from the top and bottom views of the freeze-dried PC_2_I_2_H hydrogel film, which have been exposed to ambient temperature for more than 6 months, are shown in Fig. S9.† Interestingly, the micro-network structures as well as the porous structure of the PC_2_I_2_H hydrogel still retain their shape and remain stable after several months. In summary, SEM analysis showed the formation of porous networks of the hydrogels as well as the micro-network structures located on the surface of the hydrogels.

### 3.4 TEM analysis

TEM analysis was used to study the structural properties of the O-MWCNTs and flakes, and the structure of the hydrogel network. Fig. 5 shows the TEM images of as-synthesized PC_2_I_2_H film sample (prepared by ultramicrotome) as well as the O-MWCNTs, as control. As shown in Fig. 5a–d, the O-MWCNTs have retained their original structure after oxidation process. However, the defects were observed on the walls of some O-MWCNTs (marked with red circles in Fig. 5c and d). These defects may be due to the inherent defects of the carbon nanotubes or may be created by the oxidation treatment. In addition, degradation starts from defective sites on the O-MWCNTs.^52^ The defects on CNTs increase their reactivity and can act as attackable sites in CNT degradation.^53,54^Fig. 5e–h clearly shows the flakes produced from O-MWCNTs degradation inside the hydrogel network in the size range of 10–100 nm. These O-MWCNT flakes containing carboxyl and hydroxyl groups (oxygen-containing functional groups) were able to crosslink PAM chains by forming hydrogen bonds with amide groups to form a hydrogel network. Fig. 5e shows the individual flakes that have uniformly distributed in the hydrogel film network. Whereas the smaller flakes are observed as repetitive patterns of the multiple batches in the structure of the hydrogel (Fig. 5f). This may be because smaller flakes contain more carboxyl groups and can easily form hydrogen bonds with other flakes, leading to cluster formation. The O-MWCNT flakes detected in both morphologies, including individual distribution and multiple patterns, are able to form physically crosslinked PAM networks. Some incompletely degraded O-MWCNTs were also found in the PC_2_I_2_H hydrogel film as shown in Fig. 5g and h.

### 3.5 Mechanical properties of the hydrogel films

**Fig. 6:**
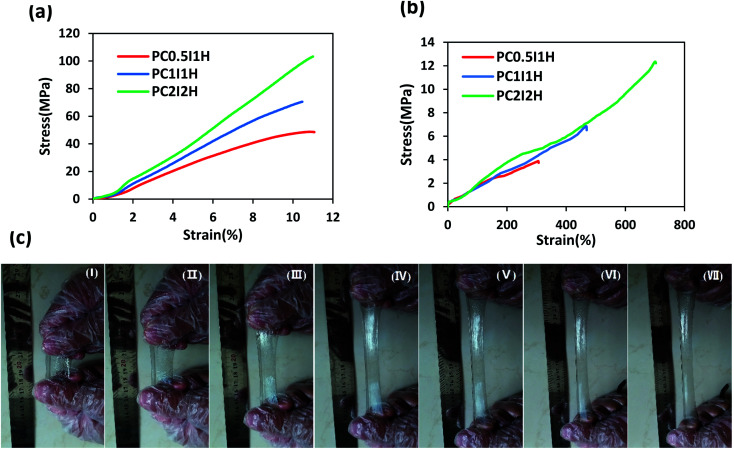
(a) Tensile stress–strain curves of as-synthesized hydrogel films, (b) tensile stress–strain curves of swollen hydrogel films, (c) photographs of PC_2_I_2_H hydrogel during stretching.

The mechanical properties of as-synthesized hydrogel films and swollen hydrogel films were evaluated by tensile tests. Fig. 6a shows the tensile stress–strain curves for as-synthesized hydrogel films. In addition, various tensile parameters, including Young's modulus, tensile strength, and strain at breaking, are listed in Table S4.† The stress–strain curves of different hydrogels showed an increasing trend in the tensile parameters with increasing the O-MWCNTs content from 0.04 wt% to 16 wt% (Fig. 6a and Table S4†). For example, PC_2_I_2_H hydrogel showed an increase more than 150% in Young's modulus as well as 210% in tensile strength compared with PC_0.5_I_1_H hydrogel. In the network of the hydrogel films, in addition to the flakes as physical crosslinking agents, free O-MWCNTs are also present as confirmed by Raman and TEM analyses. These free O-MWCNTs can act as fillers in the network and improve the mechanical properties of the hydrogel. Therefore, by increasing the feed amount of the O-MWCNTs (*i.e.* increasing the flakes content), the density of crosslinks and the content of the O-MWCNTs fillers in the hydrogel network increase and as a result, the mechanical properties of the hydrogel films are improved. It has been indicated that the incorporation of CNTs as a filler to the polymer matrix as well as strong hydrogen bonding in the hydrogel network using O-CNTs, can enhance the mechanical performance of nanocomposites.^55–57^ Studies have also indicated that the presence of the embedded micro-network structures in hydrogels can increase the mechanical properties of the hydrogels.^41,43^ Therefore, the micro-network structures observed on the outer surface of the hydrogel films (especially with higher density in PC_2_I_2_H hydrogel) may also affect their mechanical properties (Fig. S8†).

To further investigate the mechanical properties of the hydrogel films, the tensile test of the swollen hydrogel films was also performed. Fig. 6b shows the tensile stress–strain curves of swollen hydrogel films. The tensile parameters of the swollen hydrogels are summarized in Table S5.† According to the data in Table S5,† the value of all the tensile parameters of the hydrogels increased with increasing the O-MWCNT content in the hydrogel synthesis. For example, PC_2_I_2_H hydrogel showed the highest tensile strength, Young's modulus and strain at breaking compared with the other two hydrogel films. This may be because as the O-MWCNT feed content increases (*i.e.* increasing the flakes content), the crosslinking density as well as the number of hydrogen bonds in the hydrogel network increases. Compared with as-synthesized hydrogel films, swollen hydrogels exhibited an increase in strain at breaking, as well as a significant reduction in the Young's modulus and tensile strength (Fig. 6a and b). Due to the mobility of the network chains resulting from the water infiltration into the as-synthesized hydrogels, the stretchability increases while the tensile strength decreases. However, the PC_2_I_2_H hydrogel film indicated even better mechanical properties than some chemically crosslinked PAM hydrogels.^58,59^ A comparison of the mechanical properties of the hydrogel films with some of the previously synthesized PAM hydrogels is presented in Table 3. The content of crosslinkers and initiators, various synthesis methods, as well as defects in the hydrogel network are among the factors that lead to the reporting of different values for the mechanical properties of PAM hydrogels^58–64^

**Table 3:** A comparison of the mechanical properties of the hydrogel films with some PAM hydrogels

Samples	Tensile strength (kPa)	Young's modulus (kPa)	Elongation at break (%)	Ref.
PAM	8.4	3.9	254	59
PAM	115	21	700	64
PAM	0.16	—	134	58
PAM	110	190	85	62
PAM	23.6	—	—	61
PAM	68.2	22	707	63
PC_2_I_2_H^a^	12.35	1.88	703	This work
PC_2_I_2_H^b^	103.3 (MPa)	913 (MPa)	11.01	This work

a Swollen hydrogel.

b As-synthesized hydrogel.

Fig. 6c shows the photographs of the excellent stretchability of PC_2_I_2_H hydrogel at different intervals. Using hand force, the hydrogel is stretched about 6 times its original length. In addition, the PC_2_I_2_H hydrogel with small notches on the edges (created by scissors) showed high stretchability and tear resistance in the tensile test (Video S2†). This may be due to the mechanisms of energy-dissipation and stress-distribution in the hydrogel.^65,66^ The results of the tensile tests showed that PC_2_I_2_H hydrogel has the best mechanical properties compared with other hydrogel films due to its evolved network structure (in agreement with SEM results).

### 3.6 TGA studies of the nanocomposite hydrogel films

**Fig. 7:**
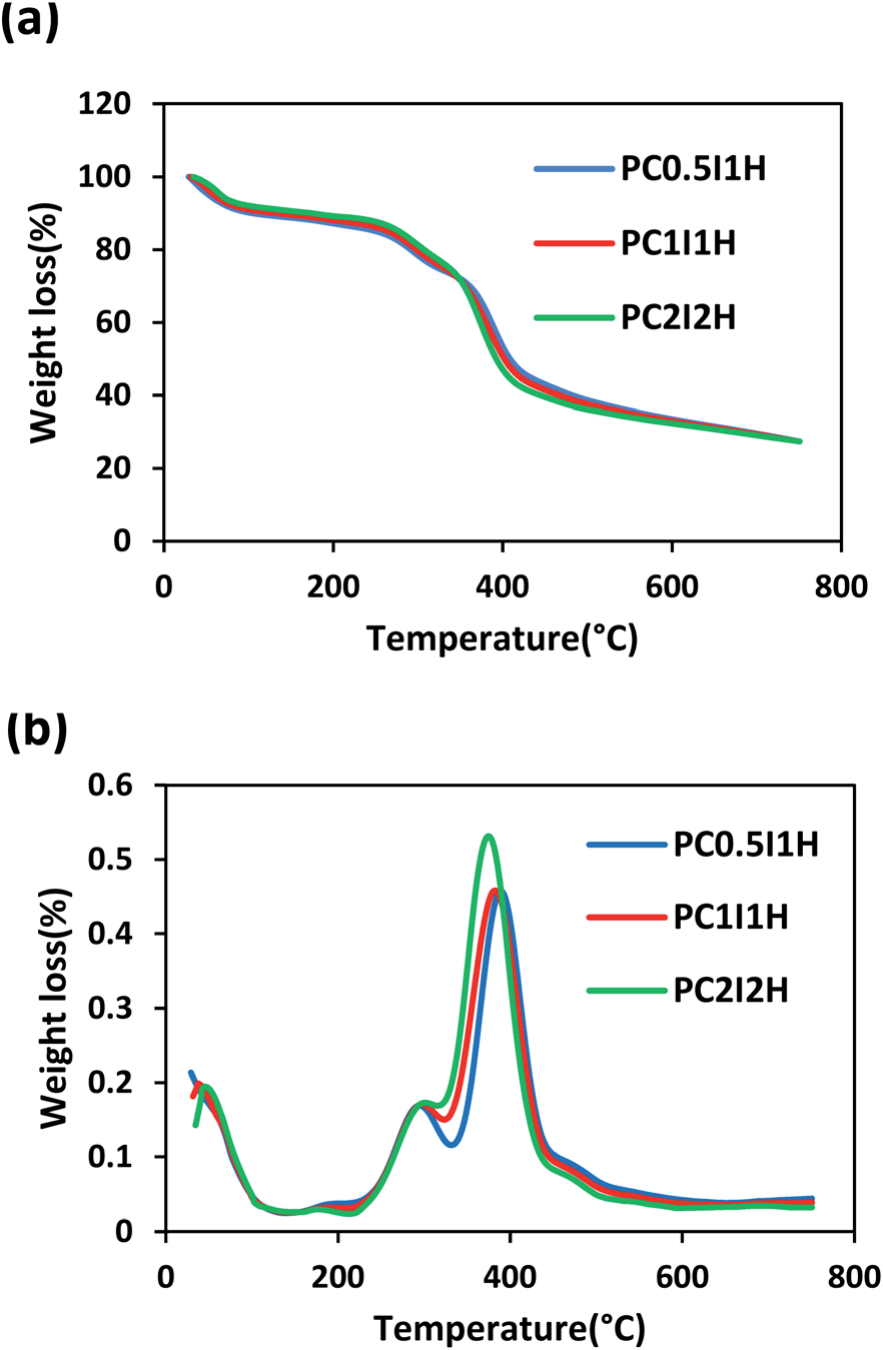
(a) The TGA curves of the hydrogel films, (b) The DTG curves of the hydrogel films.

Thermal stability of the freeze-dried hydrogel films was examined by thermogravimetric analysis (TGA). Fig. 7a shows the TGA curve of the hydrogel films, which are very similar. Overall, the weight loss of the hydrogel films in the temperature range of 30–750 °C can occur in three stages as follows: the first stage is related to the evaporation of water molecules in the hydrogel (30–230 °C); the second stage can be attributed to the removal of different functional groups (*e.g.*, COOH, OH, NH_2_) from the hydrogel network (230–350 °C); and the final stage is attributed to the decomposition of the main structure of the hydrogel (350–750 °C). By increasing the O-MWCNT feed content (*i.e.* increasing flakes content), the degradation trend of the hydrogel films was unexpectedly reversed when the temperature exceeded 350 °C (Fig. 7a). For example, PC_0.5_I_1_H with the lowest flakes content showed the highest thermal stability (Table S6†). However, with increasing temperature up to 750 °C, the weight loss in the hydrogels became approximately equal (Table S6†). This may be because the thermal decomposition of PAM chains of the hydrogels is associated with the formation of thermally stable imide species. In addition, the presence of the carboxyl groups on the O-MWCNT flakes/free O-MWCNTs may prevent the formation of the imide groups.

To further study the thermal properties of the hydrogel films, the DTG curve was used as shown in Fig. 7b. Two prominent peaks in the DTG curves are attributed to two main regions of thermal degradation between 200 and 500 °C. Hence, the first and second stages of thermal degradation are denoted as *T*_max1_ and *T*_max2_, respectively (*T*_max_ is the temperature at which maximum weight loss occurs). The decreasing trend of *T*_max1_ in the hydrogel films is as follows: PC_2_I_2_H > PC_1_I_1_H > PC_0.5_I_1_H. This may be due to the accurate crosslinking of PAM chains by the O-MWCNT flakes. In addition, free O-MWCNTs in the hydrogel network can act as heat distributors.^67^ Therefore, an increase in the density of crosslinks in the hydrogels network leads to an increase in *T*_max1_. In contrast to *T*_max1_, *T*_max2_ shows a reverse decreasing trend for the hydrogel films. At 750 °C, the hydrogel films remain as char with a similar residual weight. The formation of this char may be due to the decomposition of O-MWCNTs and their derivatives as well as stable imide species. A summary of TGA results for the hydrogel films is presented in Table S6.†

### 3.7 Swelling studies

**Fig. 8:**
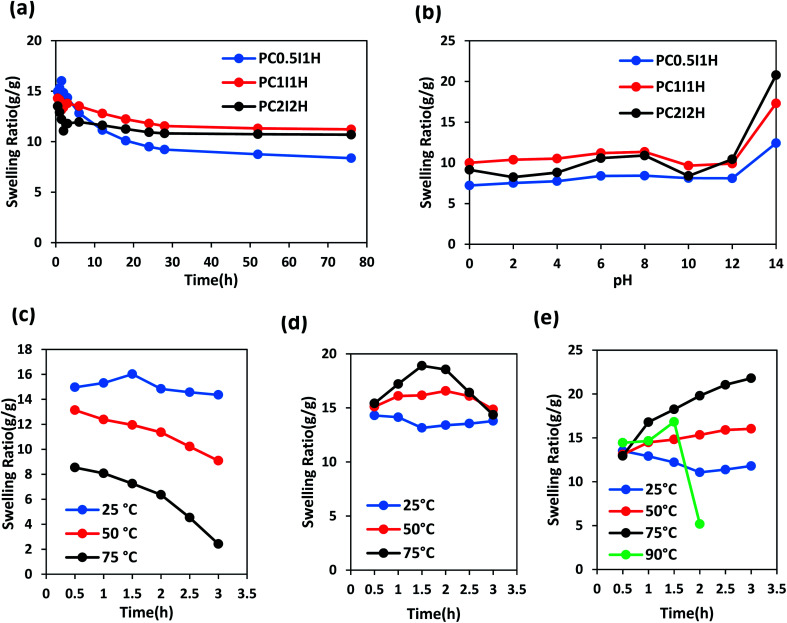
The swelling ratio curves of different as-synthesized hydrogel films in distilled water at various conditions. (a) At room temperature during 76 h, (b) at different pH values after 76 h, (c–e) at different temperatures during 3 h for PC_0.5_I_1_H, PC_1_I_1_H and PC_2_I_2_H samples, respectively.

Fig. 8a shows the swelling curves of different as-synthesized hydrogel films in distilled water at room temperature during 76 h. A sharp peak is observed in the swelling pattern of the PC_0.5_I_1_H hydrogel after 1.5 h of swelling. This peak may be due to residual impurities in the hydrogel (*e.g.*, unreacted AM monomers and NaPS residues), resulting in a concentration difference between the hydrogel network and external solution. Therefore, due to the osmotic pressure created, water molecules move into the hydrogel network, which eventually leads to rapid and temporary swelling. Similar peaks are also observed in the hydrogel films with high NaPS content (Fig. S4†). In contrast, PC_1_I_1_H and PC_2_I_2_H hydrogels show a drop in the swelling pattern (with higher intensity for PC_2_I_2_H) in the range of 0.5–3 h (Fig. 8a and S5†). This phenomenon may be attributed to the hydrophobicity of the hydrogel films due to the presence of inherently hydrophobic free O-MWCNTs in network. As swelling continues, the hydrogels reach their initial equilibrium swelling after about 3 h (PC_0.5_I_1_H and PC_1_I_1_H) and 6 h (PC_2_I_2_H). The order of swelling ratios in different hydrogel films in the first 3 h after swelling is as follows: PC_0.5_I_1_H >PC_1_I_1_H> PC_2_I_2_H. This decreasing order may be attributed to the density of the crosslinking in the hydrogel films. As the content of the O-MWCNT flakes increases, the crosslinking density of the hydrogel increases and thus the network swelling capability decreases. The density of crosslinks (created by the O-MWCNT flakes) increases with increasing the content of O-MWCNT; as a result, the hydrogel swelling ratio decreases. The swelling ratio of all hydrogels decreased after 76 h; however, it was more noticeable for PC_0.5_I_1_H film (Table S2†). This may be due to the partial dissolution of the hydrogel, which reduces the mass of the hydrogel and thus reduces the swelling ratio.^33^ Table S2† shows a summary of the results related to the swelling of different hydrogel films in distilled water during 76 h. According to Table S2,† the order of stability of different hydrogel films in distilled water is as follows: PC_2_I_2_H > PC_1_I_1_H > PC_0.5_I_1_H. PC_2_I_2_H hydrogel showed maximum stability and minimum reduction in swelling ratio compared with other hydrogels due to efficient crosslinking in the hydrogel network.

Swelling behaviour of as-synthesized hydrogel films was studied at a pH range of 0–14 during 2,3 and 76 h (Fig. S10–S12† and 8b). As shown in Fig. 8b, the order of swelling ratio in different hydrogel films at pH 14 after 76 h is as follows: PC_2_I_2_H > PC_1_I_1_H > PC_0.5_I_1_H. This trend may be related to the repulsion between negative charges of carboxylate groups resulting from the hydrolysis of carboxyl groups on O-MWCNT flakes (also free O-MWCNTs) in the hydrogel network (as well as hydrolysis of a large number of amide groups from PAM). In addition, compared with PC_0.5_I_1_H and PC_1_I_1_H hydrogels, the high stability of PC_2_I_2_H hydrogel at pH 0 is likely due to the higher density of crosslinks as well as the accurate crosslinking in the hydrogel network. As shown in Fig. 8b, the swelling of the PC_2_I_2_H hydrogel shows the highest pH sensitivity compared with the other two hydrogels. This is another evidence for the stable structure of the PC_2_I_2_H hydrogel network. In addition, due to the weak crosslinked network (unstable structure), PC_0.5_I_1_H hydrogel showed the greatest changes in the swelling ratios at different pH during 2, 3 and 76 h (Fig. S10†). The swelling ratios of the hydrogel films at different pH values after 76 h are given in Table S7.†

To evaluate the stability of the as-synthesized hydrogel films at different temperatures, their swelling behaviour was studied during 3 h. Fig. 8c shows the swelling curves of PC_0.5_I_1_H hydrogel film in distilled water at temperatures of 25, 50 and 75 °C in the range of 0.5–3 h. As the temperature rises from 25 °C to 75 °C, the swelling ratio of PC_0.5_I_1_H hydrogel decreases. In addition, the slope of the swelling isotherm is higher at higher temperatures during 3 h. For example, the greatest reduction in swelling was observed after leaving the PC_0.5_I_1_H hydrogel in distilled water at 75 °C for 3 h. Low crosslinks density and weak interactions in the PC_0.5_I_1_H hydrogel may cause the hydrogel network to not show much resistance to heat energy and to collapse. In addition, part of the hydrogel may be dissolved in the warm water due to weak interactions between network and the O-MWCNT flakes.^33^

Fig. 8d shows the swelling curves of PC_1_I_1_H hydrogel in distilled water at different temperatures during 3 h. Based on Fig. 8d, the swelling curve pattern of PC_1_I_1_H hydrogel shows an upward trend at temperatures of 50 and 75 °C in the first 1.5 h of swelling. Increasing the temperature increases the movement of water molecules and accelerates the flow of water into the hydrogel network, and ultimately increases swelling. In addition, increasing the temperature may also accelerate the hydrolysis rate of the hydrogel network. In the second 1.5 h of swelling, the swelling curves of PC_1_I_1_H hydrogel at high temperatures, especially at 75 °C, exhibit a significant reduction in the swelling over time (Fig. 8d). Weak crosslinks in the hydrogel network may be gradually broken under heat energy. This causes the porous structure of the hydrogel to collapse, which eventually reduces swelling.

PC_2_I_2_H hydrogel film exhibited significant stability in distilled water at different temperatures as shown in Fig. 8e. During 3 h at 25 °C, the swelling curve of PC_2_I_2_H hydrogel initially shows a decreasing trend due to the hydrophobic CNT-based hydrogel. As swelling continues, the PC_2_I_2_H hydrogel reaches initial equilibrium swelling. The swelling of PC_2_I_2_H hydrogel at temperatures of 50 and 75 °C is more than that at 25 °C. This may be due to the increased movement of water molecules with increasing temperature and/or the increase in the volume of the hydrogel pores (resulting from the breaking of weak physical crosslinks in the network under thermal energy). As shown in Fig. 8e, by increasing the temperature from 25 to 75 °C, the hydrogel swelling isotherms show an upward trend with a steeper slope at 75 °C. In addition, by raising the temperature to 90 °C, the swelling of the hydrogel gradually increases to 1.5 h (but is less than swelling at 75 °C), then after 0.5 h, the swelling drops sharply. The swelling of PC_2_I_2_H hydrogel at 90 °C may be interpreted as follows: the maximum swelling (compared with other temperatures) in the first 0.5 h is attributed to the high kinetic energy of water molecules; high thermal energy at 90 °C causes rapid hydrolysis and a decrease in hydrogel mass (the second 0.5 h); breaking a number of crosslinks in the hydrogel network increases the pores volume and increases swelling (the third 0.5 h); and finally, the collapse occurs (after 1.5 h). Excellent stability of the PC_2_I_2_H hydrogel at 90 °C for at least 1.5 h can be attributed to the strong crosslinking in the hydrogel network, whereas, both PC_0.5_I_1_H and PC_1_I_1_H hydrogel films were dissolved after immersion in water for approximately 0.5 h at 90 °C. Table S8† shows the swelling ratios of the as-synthesized hydrogel films after immersion in distilled water for 0.5 and 3 h at different temperatures. According to Table S8,† PC_2_I_2_H hydrogel shows the highest swelling and stability at high temperatures after immersion in distilled water for 3 h. A representation of the thermal stability of the PC_2_I_2_H hydrogel film is presented in Video S3.†

## 4 Conclusions

In this research, a novel and simple method for the synthesis of PAM/O-MWCNT flakes nanocomposite hydrogel films through the O-MWCNT degradation approach was reported for the first time. O-MWCNTs were degraded *in situ* into small flakes in the presence of NaPS under thermal conditions in aqueous solutions of the AM monomers. Simultaneously, polymerization of the AM occurred *in situ* using free radicals, which eventually led to the formation of the PAM chains. The O-MWCNT flakes with active oxygen-containing groups were able to form physically crosslinked hydrogel networks by forming hydrogen bonds with PAM chains. The hydrogel films could be produced using the feed amount of O-MWCNTs in the weight range of 0.04 to 0.16 wt% without using any crosslinkers. It was suggested that the production of ROS and their penetration into the O-MWCNTs walls through defect sites lead to O-MWCNTs degradation. However, some initial O-MWCNTs remained undegraded inside the hydrogel films as confirmed by TEM and Raman analyses. The presence of the O-MWCNT flakes in the hydrogel network was also confirmed by TEM. The porous structure of the hydrogel networks as well as unique micro-network structures located on the surface of the hydrogel films were observed by SEM. The results showed that the evolved PC_2_I_2_H hydrogel film has excellent swelling, thermal and mechanical properties compared with other synthesized hydrogel films. In addition, this hydrogel showed good stability in different pH and temperatures. Applying the strategy used in this work to synthesize hydrogels may also have promising results for other carbon nanomaterials.

## Author contributions

Alireza Yaghoubi performed the experimental procedures and wrote the manuscript text, Ali Ramazani and Hossein Ghasemzadeh edited the manuscript text and helped perform the experiments. All authors reviewed the manuscript.

## Conflicts of interest

There are no conflicts to declare.

## Supplementary Material

RA-011-D1RA07825A-s001

RA-011-D1RA07825A-s002

RA-011-D1RA07825A-s003

RA-011-D1RA07825A-s004
