# Supplementary material for: Synthesis of physically crosslinked PAM/CNT flakes nanocomposite hydrogel films via a destructive approach
Source: RSC Adv. 2021 Dec 7;11(62):39095–107. doi: 10.1039/d1ra07825a (PMC9044412; doi:10.1039/d1ra07825a)
Supplement: RA-011-D1RA07825A-s001 [file RA-011-D1RA07825A-s001.pdf]

Electronic Supplementary Material (ESI) for RSC Advances.
This journal is © The Royal Society of Chemistry 2021

Supporting Information

Synthesis of physically crosslinked PAM/CNT flakes nanocomposite hydrogel films via a destructive approach

Alireza Yaghoubi [1], Ali Ramazani [1, 2, *], Hossein Ghasemzadeh [3]

[1] Department of Chemistry, Faculty of Science, University of Zanjan, 45371-38791 Zanjan, Iran

[2] Department of Biotechnology, Research Institute of Modern Biological Techniques (RIMBT), University of Zanjan, 45371-38791 Zanjan, Iran

[3] Department of Chemistry, Faculty of Science, Imam Khomeini International University, 34148-96818, Qazvin, Iran

*Corresponding Author. E-mail: aliramazani@gmail.com; aliramazani@znu.ac.ir

List of contents	page
Part 1. Analysis and stability of the MWCNTs and O-MWCNTs	S3
Fig. S1. FTIR spectra of the MWCNTs and O-MWCNTs	S4
Fig. S2. TGA curves of the MWCNTs and O-MWCNTs	S5
Fig. S3. The stability of the MWCNTs and O-MWCNTs suspensions	S6
Part 2. Optimizing the amount of sodium persulfate (NaPS) for synthesis of the hydrogel films	S7
Fig. S4. Swelling curves of the hydrogel films using different amounts of NaPS	S8
Fig. S5. Part of the swelling curves in Fig. S4	S8
Table S1. The swelling parameters of different hydrogel films in distilled water during 52 h	S9
Table S2. The swelling parameters of Optimized hydrogel films in distilled water during 76 h	S9
Part 3. Preparation, analysis and characterization of the hydrogel films	S10
Fig. S6. The photographs of the preparation process of as-synthesized hydrogel films	S10
Table S3. Assignments of IR bands for the hydrogel films	S10
Fig. S7. The SEM image of the PC_3I_3 film	S11
Fig. S8. The micro-network structures of different hydrogel films	S12
Fig. S9. The SEM images of PC_2I_2H hydrogel film after 6 months from synthesis	S13
Table S4. The mechanical properties of as-synthesized hydrogel films	S14
Table S5. The mechanical properties of swollen hydrogel films	S14
Table S6. Summary of the TGA results for the hydrogel films	S14
Part 4. Stability studies of the hydrogels at different pH and temperatures	S15
Fig. S10. Swelling curves of the $PC_{0.5}I_1H$ hydrogel film in distilled water at different pH values	S16
Fig. S11. Swelling curves of the PC_1I_1H hydrogel film in distilled water at different pH values	S19
Fig. S12. Swelling curves of the PC_2I_2H hydrogel film in distilled water at different pH values	S19
Table S7. The swelling ratios of the as-synthesized hydrogel films at different pH values.	S20
Table S8. The swelling ratios of the as-synthesized hydrogel films at different temperatures	S20

Part 1. Analysis and stability of the MWCNTs and O-MWCNTs

FTIR analyze. The FTIR spectra of the pristine MWCNTs and O-MWCNTs are shown in Fig. S1. The spectra show a broad bond at about 3436cm^{-1} which is attributed to stretching vibrations of O-H groups (resulting from adsorbed water molecules and/or -OH moieties of carboxyl and hydroxyl groups on walls of nanotubes)[1]. Adsorption bands located at 2922 and 2856 cm-1 are corresponded to asymmetric and symmetric stretching of C–H bonds (from -CH_2), respectively. These bands are observed with higher intensities in the O-MWCNT spectrum. This may be due to the formation of large contents of carbon with sp3 hybridization during oxidation treatment. In addition, after performing the oxidation process on the MWCNTs, the peaks at 1726 and 1580 cm-1, which are related to carbonyl(C=O) groups (from COOH) and asymmetric -COO- stretch, significantly strengthened[2]. This indicates successful oxidation of the MWCNTs by acid mixture (H_2SO_4/HNO_3,3:1). Other peaks in the O-MWCNT spectrum are observed at 1413,1389 and 1026 cm-1, which can be attributed to CH_2 bending, C-OH bending and C-O stretching vibrations, respectively[3]. Therefore, FTIR analysis indicated that oxidation by the acid mixture treatment has been able to produce many oxygen-containing functional groups, especially carboxyl, on walls of the nanotubes, which significantly increased the hydrophilicity of the nanotubes (Fig. S3).

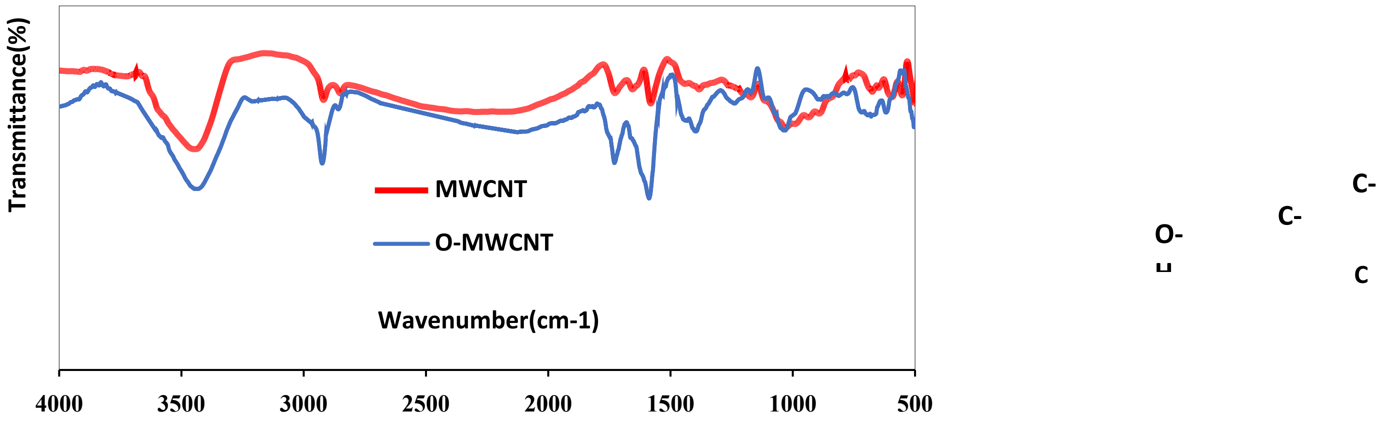

Fig. S1 FTIR spectra of the MWCNT and O-MWCNT.

TGA analyze. The thermal stability of the pristine and oxidized MWCNTs were evaluated by TGA as shown in Fig. S2. In addition, the amount of oxygen-containing functional groups such as carboxyl and hydroxyl formed on the nanotube walls was estimated through TGA profiles. The weight loss of the MWCNTs and O-MWCNTs as a function of temperature occurs in several steps as follows: [4] the first step is between room temperature and 150 ° C, which is related to the evaporation of water molecules adsorbed on the surfaces of nanotubes; the second stage in the range of 150 to 500 ° C is attributed to the detachment of carboxyl and hydroxyl groups from the surfaces of the nanotubes, in this range, weight loss was estimated about 2.74% for pristine MWCNTs, while it was 10.35% for O-MWCNTs, indicating the correct implementation of the oxidation process; the final stage of weight loss occurs at temperatures above 500 ° C where the nanotubes decompose structurally. Thermal decomposition of O-MWCNT begins earlier than that of MWCNT (Fig. S2), which results in a drop of about 70 ° C in T_{50} (temperature at which nanotubes lose half their original

weight) after oxidation on the MWCNTs. This significant difference in the reduction of thermal stability may be due to defect formation in the nanotubes during the oxidation process.

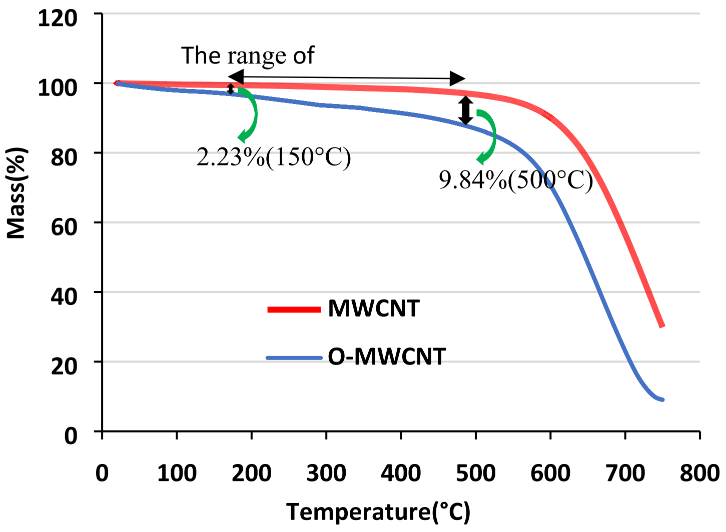

Fig. S2 TGA curves of the MWCNT and O-MWCNT (total content of functional groups, especially carboxyl and hydroxyl groups. formed on the nanotubes surfaces in the oxidation process was estimated to be about 7.61%).

The suspension stability. Fig. S3 shows the stability photographs of the MWCNTs and O-MWCNTs suspensions at different times after their intense sonication in distilled water for 0.5 h. Upon the completion of sonication, pristine MWCNTs in the suspension begin to form agglomerates and settle to the bottom of the container when their weight becomes heavy enough over time. This is due to the strong van der Waals attractions forces between the pristine MWCNTs, which causes them to agglomerate. In contrast, the O-MWCNT suspensions showed considerable stability due to the presence of hydrophilic oxygen-containing functional groups on the O-MWCNTs surfaces, which endow the nanotubes hydrogen bonding ability and hydrophilicity.

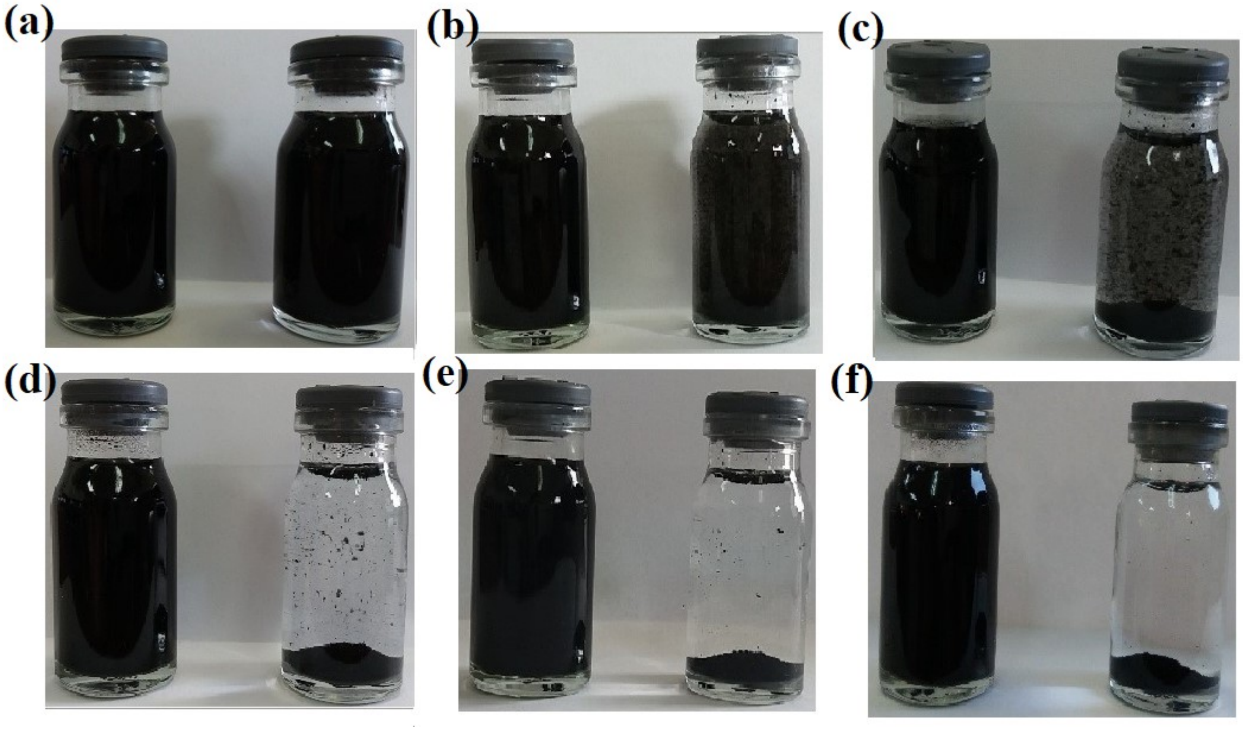

Fig. S3 The stability of the O-MWCNTs and MWCNTs (left and right glass bottles in each photograph, respectively) suspensions at different times after 0.5 h sonication in distilled water: (a) Immediately after sonication, (b, c) after 12 and 37 min, respectively, (d) after 20 h, (e, f) after 8 and 28 days, respectively.

Part 2. Optimizing the amount of NaPS for the synthesis of the hydrogel films

To optimize the amount of initiator for the synthesis of the hydrogels, different amounts of NaPS were used ranging from 0.001 to 0.01g. In addition, the amount of O-MWCNT and acrylamide (AM)monomer were held constant at 0.001 and 1.2 g, respectively. To investigate the influence of the amount of the NaPS initiator on the swelling behavior of the as-synthesized hydrogel films, 0.1 g of the hydrogel sample was immersed into distilled water to swell at room temperature. At different time intervals, swollen samples were taken out of the water and weighed after removing excess water. The swelling ratio (SR) of the hydrogel samples were calculated at different intervals according to the following formula:

$$SR = \frac{Ws - Wd}{Wd} \quad (1)$$

Where Ws and Wd are the weight of the swollen and dry hydrogels, respectively.

The swelling ratios of different hydrogel films in distilled water during 52 h are shown in Fig. S4.

The decrease in the swelling ratio of the hydrogels in the range of 0.5-52 h (76 h) was calculated according to the following formula:

$$\text{Decrease in swelling ratio (\%)} = \frac{SR_{0.5h} - SR_{52h(76h)}}{SR_{0.5h}} \times 100 \quad (2)$$

Where $SR_{0.5h}$and $SR_{52h(76h)}$ are the swelling ratios after 0.5 and 52 h (76 h).

According to Table S1, compared with other hydrogels, PC_1I_1H hydrogel showed the highest stability along with the lowest decrease in swelling ratio and the highest swelling ratio after

52 h. Therefore, since the amount of NaPS used in PC_1I_1H was equal to O-MWCNT, the optimal amount of NaPS equivalent to the amount of O-MWCNT was selected for the synthesis of subsequent hydrogel films.

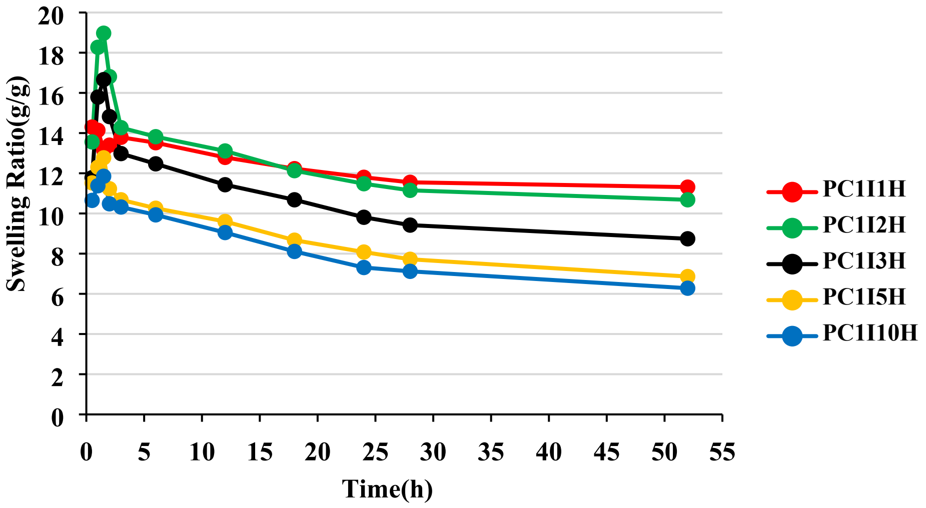

Fig. S4 Swelling ratios of different hydrogel films in distilled water.

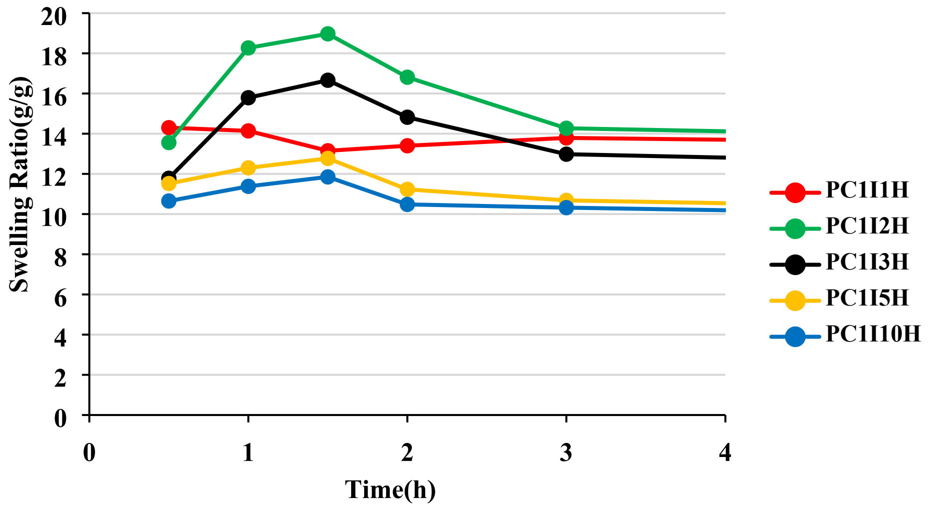

Fig. S5 Part of the swelling curves of the hydrogel films in Fig. S4 in the range of 0.5-4 h with a high magnification.

Table S1 Swelling ratio and decrease in swelling ratio of different hydrogel films.

Hydrogel film	Swelling ratio after 0.5 h	Swelling ratio after 52 h	Decrease in swelling ratio (%)
**PC_1I_1H**	14.3	11.31	20.91
**PC_1I_2H**	13.56	10.68	21.24
**PC_1I_3H**	11.78	8.74	25.81
**PC_1I_5H**	11.52	6.86	40.45
**$PC_1I_{10}H$**	10.65	6.28	41.03

Table S2 Parameters related to swelling ratios of optimized hydrogel films in distilled water during 76 h.

Part 3. Analysis and characterization of the nanocomposite hydrogel films

Hydrogel film	$PC_{0.5}I_1$	PC_1I_1	PC_2I_2
Swelling ratio after 0.5 h	14.97	14.3	13.51
Swelling ratio after 76 h	8.37	11.22	10.69
Decrease in swelling ratio (%)	44.09	21.54	20.87
Initial equilibrium swelling ratio	14.36(3 h)	13.79(3 h)	11.95(6 h)
Swelling ratio at Sharp/drop point	16.02(1.5 h)	13.15 (1.5 h)	11.07(2 h)

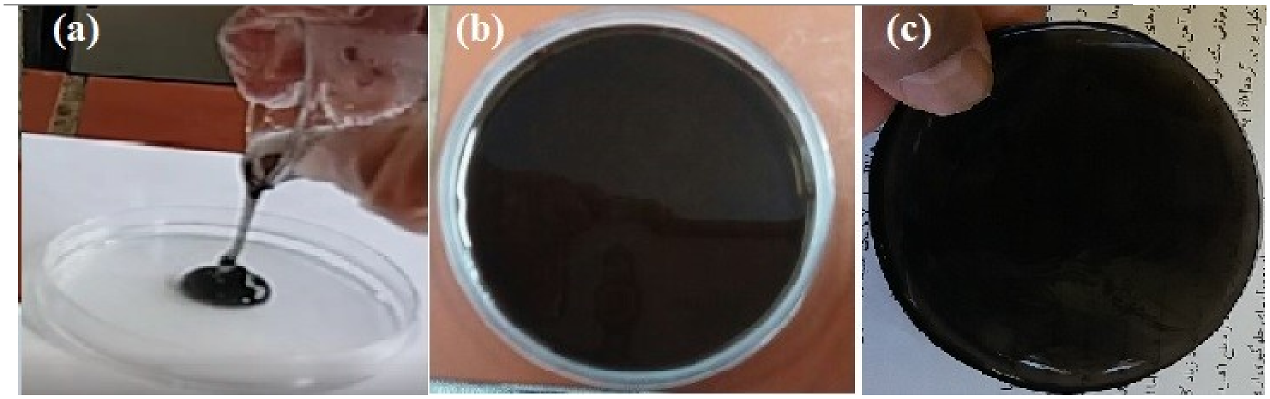

Fig. S6 The preparation process of the hydrogel films. (a) Pouring primary gel into a Petri dish, (b) molded hydrogel and (c) hydrogel film.

Table S3 Assignments of IR bands (cm^{-1}) related to the hydrogel films.

$PC_{0.5}I_1H$	PC_1I_1H	PC_2I1_2H	vibrations
3331	3329	3338	NH_2 asy stretching
N.V.	N.V.	N.V.	Conjugated NH_2
3184	3186	3190	NH_2 sy stretching
2929	2929	2926	CH_2 stretching
2870	2865	2854	CH stretching
1651	1649	1655	C=O stretching
1606	1606	1612	NH_2 bending
1446	1446	1448	CH_2 bending
1412	1412	1414	C-N stretching
1344	1344	1348	CH_2 wagging
1319	1317	1319	C-H bending
1182	1188	1186	Polymer skeletal
1117	1120	1122	C-C stretching
N.V.	N.V.	N.V.	N.V.
804	779	795	HRC-C bending
631	629	627	NH_2/OCN bending

N.V.: Not visible

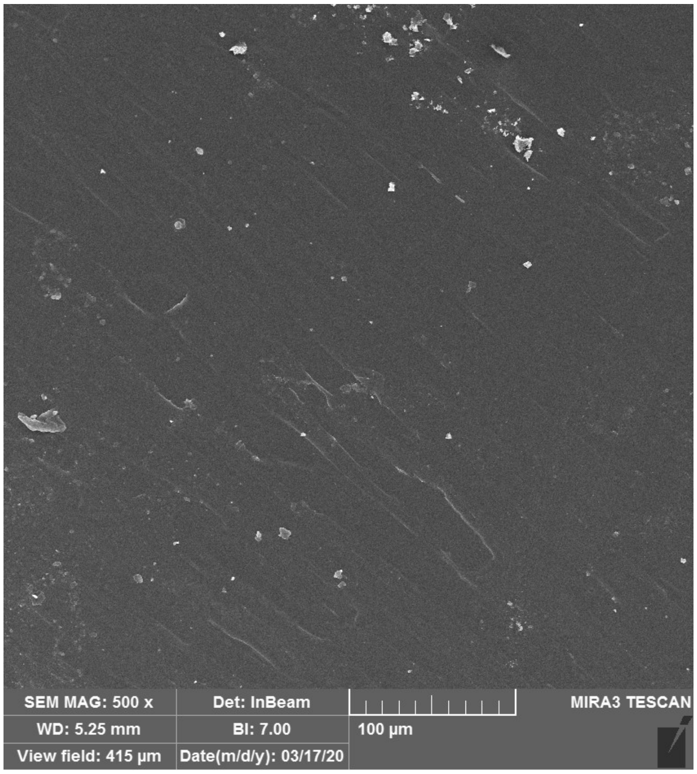

Fig. S7 The SEM image of the PC_3I_3 film with a 500x magnification.

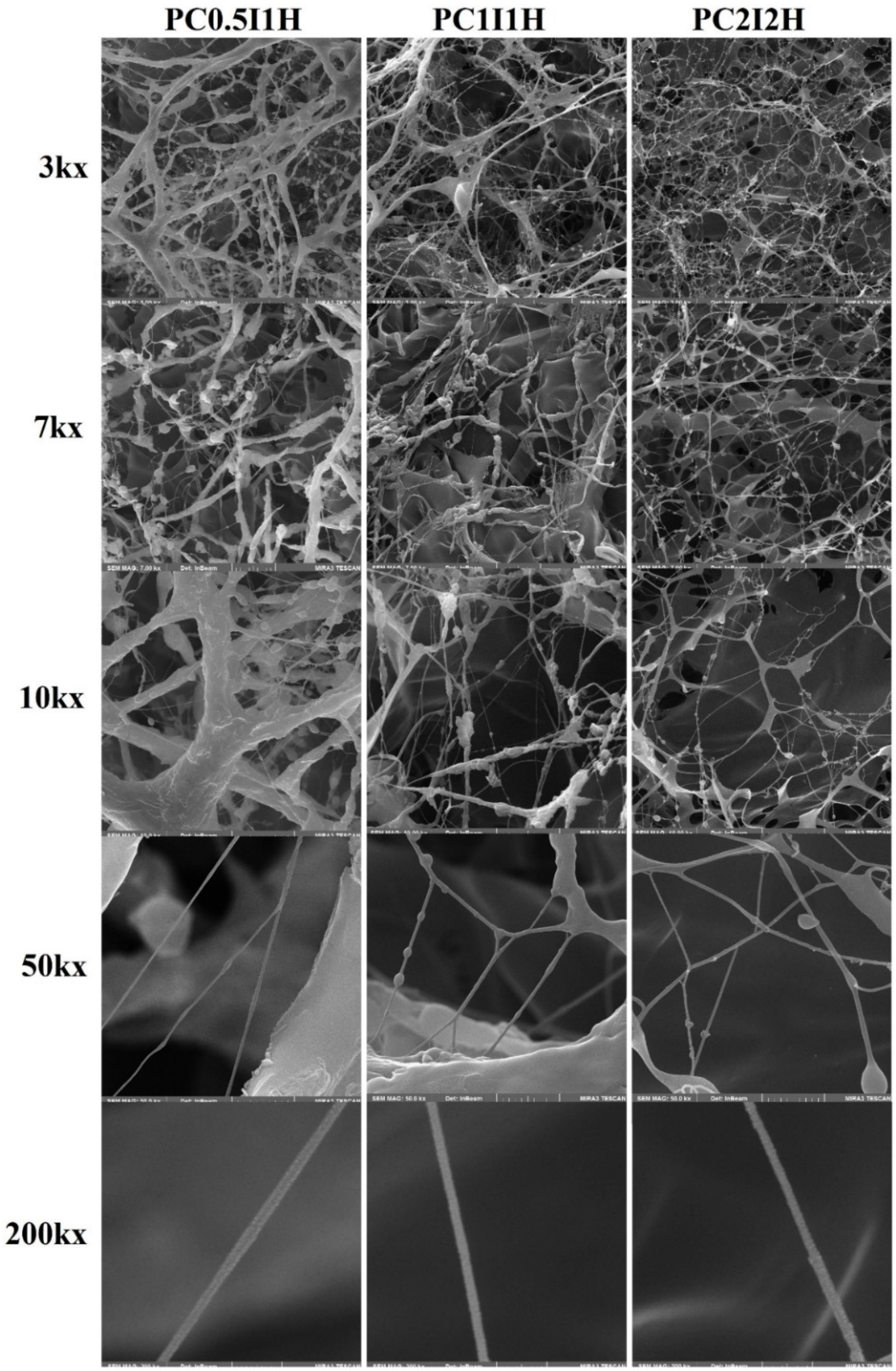

Fig. S8 The enlarged images of the freeze-dried hydrogel films (Fig.3d-f) with different magnifications. (3kx,7kx and 10 kx) The annular microstructures of (Fig. 3d and e) and micro-network structures of (Fig. 3f), (50kx) the various interactions of micro-network fibres, including helical, cross and branched, (200kx) the SEM micrographs of the micro-network fibres having approximately the same diameter.

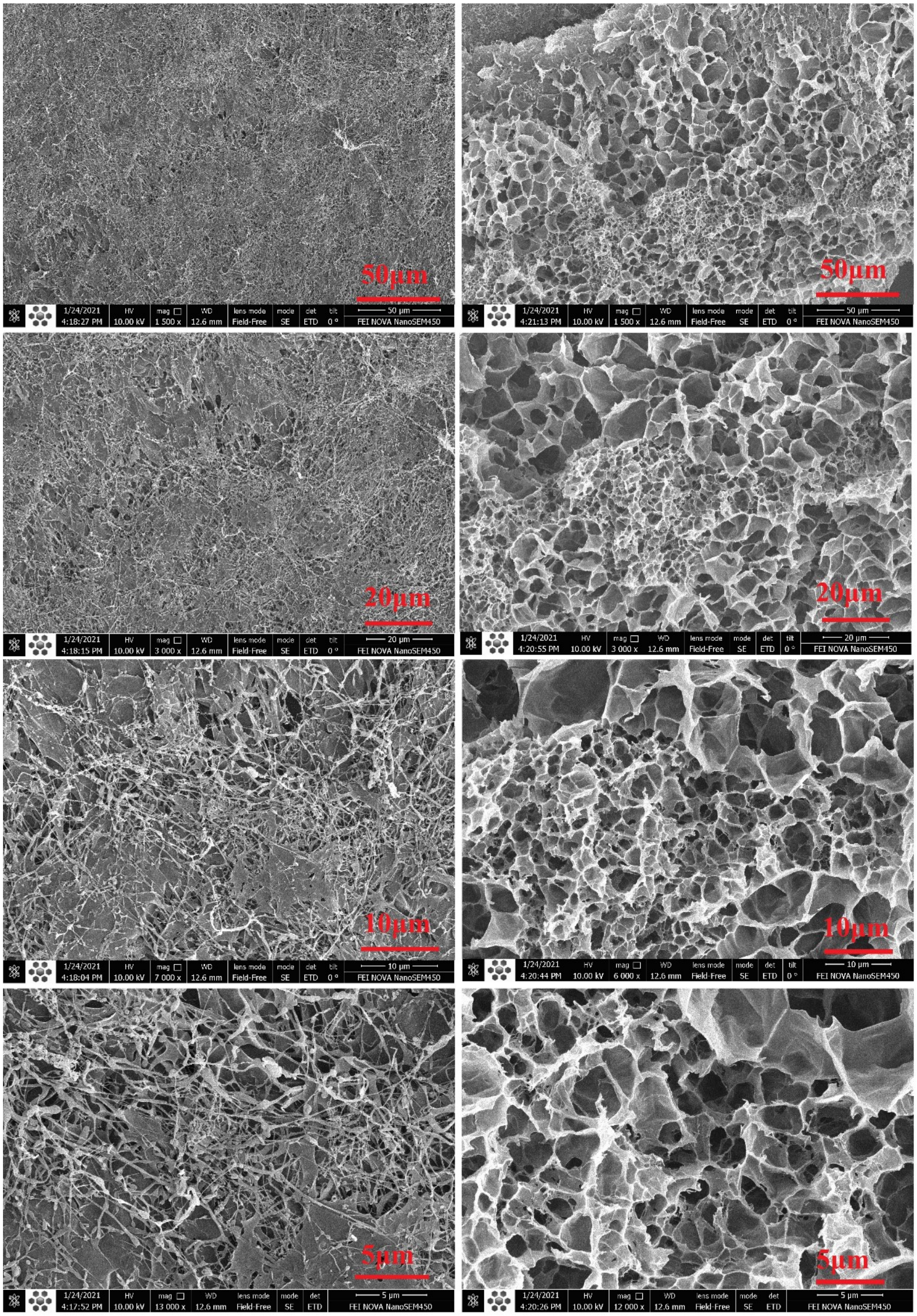

Fig. S9 The SEM images from the top view (left images) and bottom view (right images) for the freeze-dried PC_2I_2H film after 6 months from synthesis.

Table S4 The mechanical properties of as-synthesized hydrogel films in dry mode.

Samples	Tensile strength (MPa)	Young's modulus (MPa)	Elongation at break (%)
**$PC_{0.5}I_1H$**	48.7±3.6	591±20	11.12±2.49
**PC_1I_1H**	70.4±4.8	772±22	10.39±2.38
**PC_2I_2H**	103.3±3.9	913±21	11.01±2.04

Table S5 The mechanical properties of the swollen hydrogel films.

Samples	Tensile strength (kPa)	Young's modulus (kPa)	Elongation at break (%)
**$PC_{0.5}I_1H$**	3.89±0.88	1.36±0.09	307±69
**PC_1I_1H**	6.86±1.33	1.52±0.08	469±91
**PC_2I_2H**	12.35 ±1.96	1.88±0.08	703±111

Table S6 A summary of the TGA results for the hydrogel films.

Sample	Weight loss (%)			Degradation T_{max} (°C)		Residual weight (%)
	30-230(°C)	230-350 (°C)	350-750(°C)	T_{max1}	T_{max2}	
**$PC_{0.5}I_1H$**	13.92	14.09	44.60	294.92	389.21	27.39
**PC_1I_1H**	12.80	15.63	44.14	297.62	382.55	27.43
**PC_2I_2H**	11.45	16.92	44.19	301.24	374.86	27.44

Part 4. Stability studies of the hydrogels at different pH and temperatures

Swelling behavior of the as-synthesized hydrogel films at different pH. 1 M HCl and 1 M NaOH solutions were used to prepare solutions with pH 0 and 14, respectively. The solutions were also diluted with distilled water to obtain solutions with pH 2 (HCl solution) and 12 (NaOH solution). Other solutions with different pH values were prepared by diluting solutions with pH 2 and 12. Fig. S10-S12 show the swelling behavior of different hydrogel films at the pH range of 0-14 during 76 h. As shown in Fig. S10, the maximum swelling ratio of the $PC_{0.5}I_1$ hydrogel at different pH is in a neutral range (pH 6 to 8) during 2 and 3 h. This may be because the hydrogels can swell due to osmotic pressure caused by lower ionic strength of the external solution[9]. The decrease in swelling of $PC_{0.5}I_1H$ hydrogel in an acidic medium may be justified by the interaction of hydrogen bonds among carboxylic acid groups (resulting from the hydrolysis of amide groups from PAM, as well as carboxylic groups on degraded O-MWCNTs), which produces additional physical crosslinks[10]. The formation of hydrogen bonds between carboxyl groups is more likely to occur at very acidic pH values (pH<3)[9]. In addition, the interaction of H^+ ions with OH groups can also induce a decrease in concentration of ions into the network and reduce swelling [11]. At pH 4 to neutral, a large number of carboxyl groups are ionized to carboxylate groups, which increases the repulsion between negative charges and leads to the swelling of hydrogel network. The decrease of hydrogel swelling in the basic range (pH 8-14) may be due to the osmotic pressure resulting from the increase in the concentration of OH^- ions in the external solution. In addition, the charge screening effect of counter ions (Na^+) in salt solutions with pH> 9 can reduce the repulsion between negative charges of carboxylate groups. This prevents water from entering

the hydrogel and thus reduces swelling. After 76 h from immersing the $PC_{0.5}I_1$ hydrogel into distilled water, the swelling ratio decreased significantly at all pH values (except pH 14, Fig. S10). This may be attributed to the dissolution of the $PC_{0.5}I_1H$ hydrogel film in aqueous solution, which is associated with a reduction in mass (Fig. S10)[12]. In addition, the high swelling at pH 14 is due to repulsive forces between the carboxylate groups, resulting from the hydrolysis of a large number of amide groups(from PAM) and the carboxyl groups(from the O-MWCNT flakes or free O-MWCNTs) under very basic conditions [11]. After the hydrogel networks swell, a large volume of water enters the hydrogel, which leads to maximum swelling.

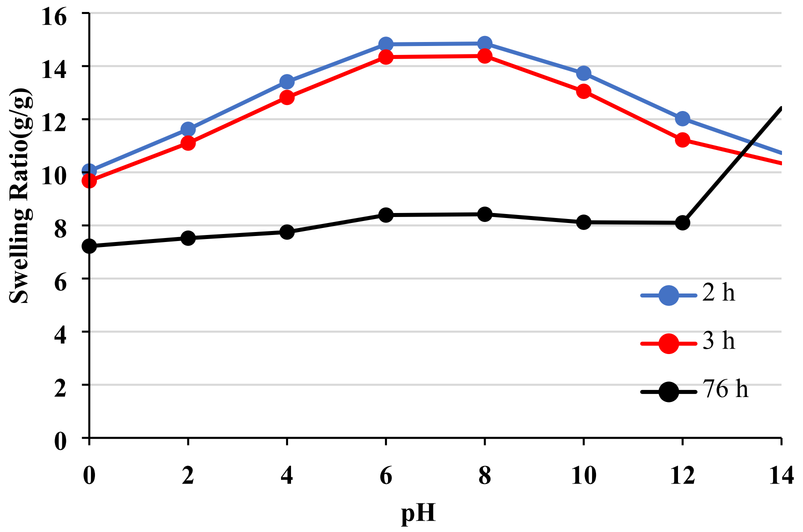

Fig. S10 Swelling curves of the $PC_{0.5}I_1H$ hydrogel film in distilled water at different pH values after 2, 3 and 76 h.

On the other hand, the swelling curves of PC_1I_1H and PC_2I_2H hydrogels (Fig. S11 and S12, respectively) in the pH range of 0-14 are different from those of $PC_{0.5}I_1H$ hydrogel, especially

at 2 and 3 h. This may be due to increased stability of the hydrogel films against dissolution by increasing the amount of the O-MWCNT flakes (as crosslinkers) in the hydrogel. For PC_1I_1H and PC_2I_2H hydrogel films, the highest swelling ratio is observed at pH 14(Fig. S11 and S12). This may be due to repulsive interactions between negatively charged carboxylate groups resulting from the hydrolyzed amide groups in the hydrogel and carboxyl groups on the flakes (as well as free O-MWCNTs). In addition, a time-dependent increasing trend in the swelling ratio is observed at pH14(Fig. S11 and S12). Under severe alkaline conditions, large amounts of amide groups in the PAM network are hydrolyzed to carboxylate groups over time. Therefore, as the density of carboxylate groups increases, the repulsion between their negative charges increases, leading to a significant increase in swelling. In addition, in alkaline hydrolysis of PAM, there is nearly alternation between amide and carboxylate groups. Under these conditions, the amide groups are partially converted to ionized carboxyl groups. The resulting negative charges repulse hydroxyl ions and do not allow hydrolysis of both adjacent amide groups. Thus, the next groups are possible to hydrolyze, which is an alternating tacticity [13]. Swelling in the pH range of 8-12 is influenced by two factors, osmotic pressure and repulsion between carboxylate groups, which act in the opposite directions (Fig. S10-S12). As pH increases from 8 to 10, the number of hydroxide ions in the solution increases, and the swelling decreases due to the osmotic pressure created by these ions. By increasing pH to 12, the number of hydroxide ions in the solution increases significantly, which can easily hydrolyze amide groups to carboxylate groups in the hydrogel network. The repulsion between negative charges of the carboxylate groups leads to swelling in the hydrogel. Swelling of different hydrogel films in the pH range of 2-6 may be further affected by osmotic pressure. As the concentration of H^+ ions in the aqueous solution increases, a

concentration difference occurs between the hydrogel and the external solution, reducing the flow of water into the hydrogel and thus reduces swelling. the swelling curves of the hydrogels at pH 0 show a time-dependent decreasing trend in swelling during 2,3 and 76 h (Fig. S10-S12). This may be due to the hydrolysis of weak crosslinks created between the flakes and the PAM chains, as well as the weight loss of the hydrogel films by releasing unreacted monomers into the solution. Decreased concentration of ions in the network under acidic conditions is another factor in reducing swelling[11].

As shown in Fig. S11 and S12, the swelling ratios in PC_1I_1H and PC_2I_2H hydrogels during 2 and 3 h at pH 0 is higher than those of other pH values (Except pH 14). This may be because some weak crosslinks in the hydrogel network are broken under severely acidic conditions, which reduces the density of the crosslinking and increases the volume of some pores in the hydrogel, and ultimately leads to a temporary increase in swelling. Whereas, the $PC_{0.5}I_1H$ hydrogel network with lower crosslinks density collapses under severe hydrolysis at pH 0, resulting in a decrease in swelling (Fig. S10). The decrease in swelling after 76 h may be attributed to the dissolution of the hydrogel films in aqueous solution.

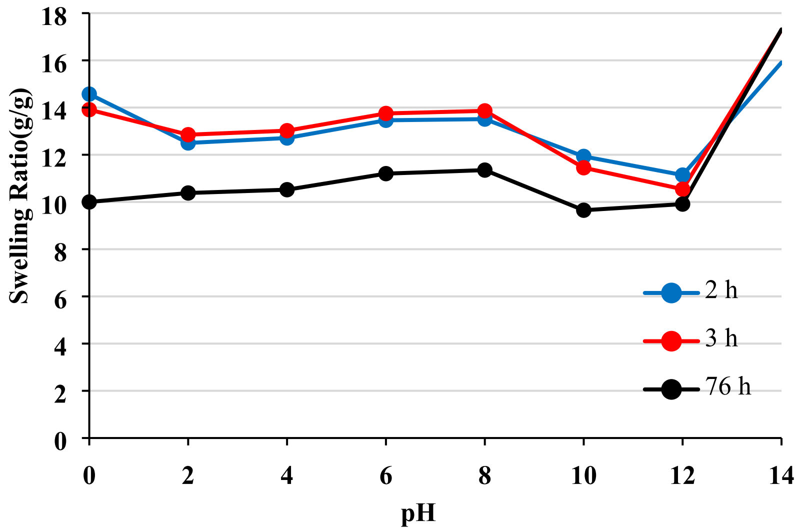

Fig. S11 Swelling curves of the PC_1I_1H hydrogel film in distilled water at different pH values after 2, 3 and 76 h.

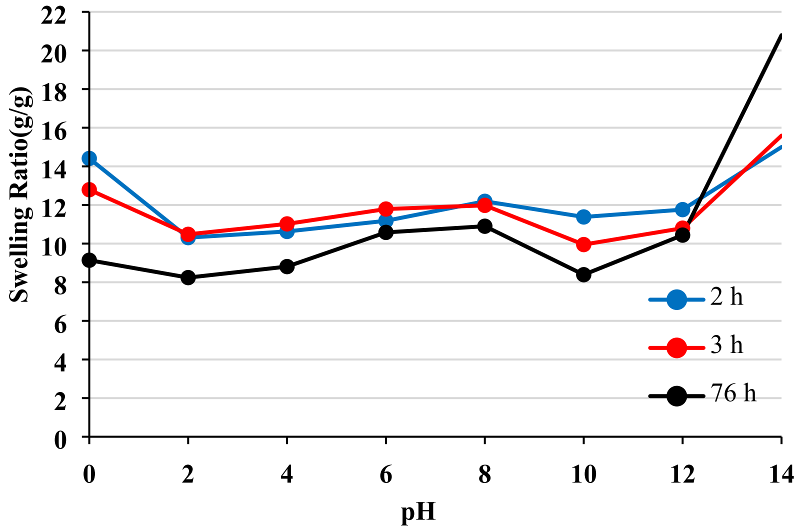

Fig. S12 Swelling curves of the PC_2I_2H hydrogel film in distilled water at different pH values after 2, 3 and 76 h.

The swelling ratios of different hydrogel films in aqueous solutions with different pH values after 76 h are given in Table S7. Compared with other hydrogel films, the PC_2I_2H hydrogel shows more changes in the swelling ratio at different pH values (Table S7).

Table S7 The swelling ratios of the as-synthesized hydrogel films at different pH values.

pH values	0	2	4	6	8	10	12	14
**$PC_{0.5}I_1H$**	7.22	7.52	7.75	8.39	8.42	8.12	8.1	12.42
**PC_1I_1H**	10.01	10.38	10.52	11.2	11.35	9.65	9.91	17.31
**PC_2I_2H**	9.14	8.24	8.81	10.58	10.9	8.39	10.44	20.79

Table S8 The swelling ratios of the as-synthesized hydrogel films at different temperatures after immersion in distilled water for 0.5 and 3 h.

**Temperature (° C)**		25	50	75	90
**$PC_{0.5}I_1H$**	0.5 h	14.97	13.14	8.54	dissolved
	3 h	14.36	9.09	2.42	-
**PC_1I_1H**	0.5 h	14.3	15.11	15.42	dissolved
	3 h	13.79	14.86	14.36	-
**PC_2I_2H**	0.5 h	13.51	13.1	12.95	14.44
	3 h	11.79	16.03	21.8	5.18(2 h)
